# Temporal Evolution of Lithium Metal Microstructures During Ultra‐High‐Capacity Stripping/Plating Cycles

**DOI:** 10.1002/advs.202506474

**Published:** 2025-06-10

**Authors:** Arghya Dutta, Yoshimi Kubo

**Affiliations:** ^1^ Center for Green Research on Energy and Environmental Materials National Institute for Materials Science 1‐1 Namiki Tsukuba 305‐0044 Japan

**Keywords:** Li deposition, Li metal battery, microstructure analysis, morphological analysis, ultra‐high‐capacity battery

## Abstract

The morphology of deposited lithium (Li) is critical to the stability and reversibility of Li‐metal batteries (LMBs). While crystallographic features of Li influence deposition morphology, the orientation of Li crystals during electrodeposition and their temporal evolution under varying kinetic and interphasial conditions remain unclear. This study investigates Li microstructures during electrodeposition at ultra‐high capacities (up to 12 mAh cm⁻^2^) and over repeated cycling, using scanning electron microscopy (SEM) and electron backscatter diffraction (EBSD). The results show that the microstructural evolution of Li depends on the interplay between interphasial properties and deposition kinetics. A layer‐by‐layer epitaxial Li growth with a coherent lattice orientation is achievable under homogeneous interphase and slow deposition kinetics. However, at higher capacities or extended cycling, deterioration of interphase homogeneity disrupts crystal matching, resulting in island‐like deposits with randomly oriented single‐crystalline grains. In contrast, an inhomogeneous interphase and faster kinetics lead to whisker‐like Li deposits. These results demonstrate that while cohesive interactions between depositing Li crystals can result in isolated single‐crystalline grains, maintaining interphase homogeneity and stability is essential to enable coherent lattice matching for layer‐by‐layer epitaxial growth. This study reveals Li microstructural evolution and offers insights for designing stable interphases and optimizing conditions for durable, high‐capacity LMBs.

## Graphical abstract

This study explores the microstructural evolution of lithium (Li) during ultra‐high‐capacity electrodeposition, emphasizing the role of interphasial properties and deposition kinetics on crystal grain orientation. Homogeneous interphase and slow deposition kinetics enable layer‐by‐layer epitaxial growth with coherent crystal grain matching, while interphase degradation disrupts crystallographic alignment, leading to island‐like Li deposits.

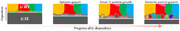

## 1 Introduction

Lithium (Li) metal is a highly promising negative electrode for next‐generation high‐energy rechargeable batteries, offering an exceptional specific capacity of 3860 mAh g⁻¹, the lowest electrochemical potential (−3.04 V vs standard hydrogen electrode (SHE)), and a low density of 0.534 g cm⁻^3^.^[^ ^1^, ^2^, ^3^ ^]^ Despite these advantages, the practical implementation of lithium metal batteries (LMBs) faces two major challenges. The first is dendritic Li growth during charging, which can cause internal short circuits and severe safety risks.^[^ ^4^, ^5^ ^]^ The second is low Coulombic efficiency (CE), resulting from non‐compact, porous Li deposits that become electrically isolated during repeated plating and stripping cycles.^[^ ^6^ ^]^ These deposits also increase side reactions with the electrolyte, leading to active Li loss.^[^ ^7^, ^8^, ^9^, ^10^ ^]^ Therefore, achieving compact, large‐sized Li deposits − or ideally, uniform, layer‐by‐layer 2D growth − is essential for realizing high‐performance LMBs.

In general, the preferred mode of electrodeposition − whether 2D layer‐by‐layer or 3D island‐like − depends on a delicate balance between thermodynamic and kinetic factors.^[^ ^11^, ^12^ ^]^ Thermodynamically, the system favors configurations that minimize the total energy during the deposition process.^[^ ^12^ ^]^ On the other side, kinetic factors such as atomic arrival rate, adatom diffusion, and nucleation‐and‐growth dynamics strongly influence the deposition pathway.^[^ ^11^ ^]^ For 2D epitaxial electrodeposition, an energetically favorable coherent lattice orientation between the electrodeposit (epilayer) and the substrate (electrode) is essential. Additionally, from a kinetic perspective, adatoms must exhibit sufficient surface mobility relative to the atomic arrival rate so that the general texture of the surface does not change and promotes a correlated orientation.^[^ ^11^ ^]^ Conversely, when the formation of the electrodeposit‐substrate interface results in an energy loss or the adatom mobility is insufficient, 3D island growth becomes the preferred mode of deposition.^[^ ^12^ ^]^ Previous studies have indeed shown that the crystallographic features are correlated with both the energetics and kinetics of Li deposition, where certain crystallographic facets demonstrate greater thermodynamic stability and kinetic preference, thereby shaping the resulting Li morphology.^[^ ^13^, ^14^, ^15^ ^]^ Therefore, gaining a clear understanding of the factors influencing the evolution of Li microstructural and crystallographic properties during deposition is imperative for controlling morphology through the manipulation of operating parameters in LMBs.

Recent studies on LMBs with solid electrolytes and capacities up to 10 mAh cm⁻^2^, have demonstrated the possibility of layer‐by‐layer Li growth, with electrodeposited grains ranging from 10 to 100 µm in width and grain boundaries predominantly oriented perpendicular to the electrode surface.^[^ ^16^, ^17^ ^]^ These findings confirm the feasibility of Li deposition with a preferred crystallographic orientation over a wide area. However, in the presence of a liquid electrolyte, the situation becomes more complex due to intricate interfacial properties. The high reactivity of Li metal with nonaqueous liquid electrolytes leads to the formation of a solid electrolyte interphase (SEI) layer, whose physical and chemical characteristics depend on the electrolyte composition.^[^ ^18^, ^19^, ^20^ ^]^ Moreover, the solvation of Li⁺ ions in liquid electrolytes, distinct from solid‐state systems, plays a critical role in governing Li deposition behavior. During Li deposition in the presence of SEI, solvated Li⁺ ions must transport through the bulk electrolyte to the interface, undergo desolvation, cross the SEI layer, and finally reduce at the electrode surface.^[^ ^18^, ^21^ ^]^ Thus, the Li⁺ ion transport properties in the bulk electrolyte, desolvation kinetics at the interface, the diffusion of Li⁺ ions, and their spatial homogeneity through the SEI are critical factors influencing the deposition patterns. While the role of SEI properties and deposition kinetics in determining bulk Li morphology has been extensively investigated, the crystallographic orientation and microstructural evolution of Li during deposition in the presence of the SEI remain insufficiently explored.^[^ ^22^, ^23^, ^24^ ^]^

In this study, we systematically investigated the temporal evolution of Li microstructure during electrodeposition at ultra‐high capacities (up to 12 mAh cm⁻^2^) and over repeated cycling, using three distinct electrolytes exhibiting varying interphasial and kinetic characteristics. To capture the structural transformations of Li deposits with high spatial and temporal resolution, we employed an advanced combination of scanning electron microscopy (SEM) integrated with electron backscatter diffraction (EBSD) and in situ optical microscopy (OM). These techniques provided critical insights into the crystallographic orientation and morphological evolution of deposited Li under different electrolyte conditions. Furthermore, to assess the interphasial properties and kinetic characteristics of the electrolytes, we performed a series of complementary analyses, including X‐ray photoelectron spectroscopy (XPS) for chemical composition evaluation, laser microscopy for surface roughness quantification, and impedance spectroscopy for interphasial kinetic measurements. The results reveal that a critical interplay between the properties of the SEI and the deposition kinetics governs the microstructural evolution of Li. A homogeneous SEI, coupled with slow deposition kinetics, promotes layer‐by‐layer 2D growth, characterized by a coherent lattice orientation, up to a moderate capacity. However, at high capacity or during extended cycling, the homogeneity of the interphase deteriorates. This leads to a transition from 2D growth to a 3D island‐like deposition, where randomly oriented single‐crystalline grains nucleate and grow in spatially separated domains. In contrast, inhomogeneous interphase, characterized by chemical and physical irregularities, combined with faster deposition kinetics, drives the formation of anisotropic whisker‐like Li deposits. Under these conditions, the Li⁺ flux becomes non‐uniform, favoring localized nucleation and rapid 1D growth of single‐crystalline whiskers. It is important to note that while both particle‐like and whisker‐like Li structures exhibit single‐crystalline features due to feasible cohesive interactions between depositing Li crystals, maintaining a stable and uniform interphase is essential for sustaining layer‐by‐layer 2D epitaxial growth with coherent lattice matching, which is key to achieving stable and high‐performance Li metal anodes. The study provides critical insights into the microstructural and crystallographic evolution of Li during electrodeposition and highlights the importance of interphasial engineering and deposition kinetics control. By optimizing electrolyte formulations, SEI properties, and operating conditions, it becomes possible to achieve stable, compact, and high‐capacity Li metal anodes, offering a clear pathway toward the practical realization of next‐generation, durable LMBs.

## 2 Results and Discussion

### 2.1 Bulk Morphologies of Li During Stripping/Plating in Different Electrolytes

**Figure 1:**
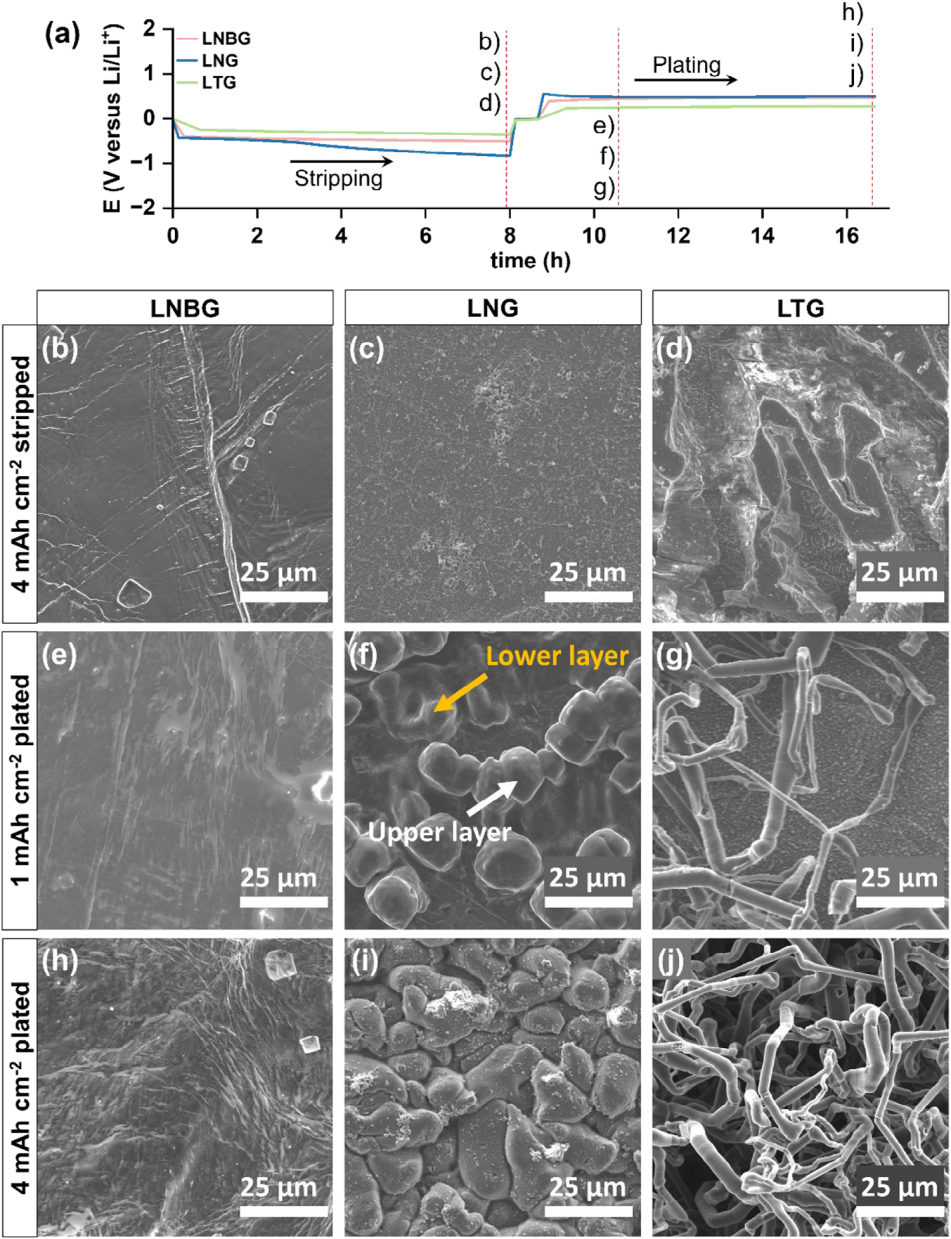
a) Galvanostatic voltage profiles for Li stripping/plating in Li|Li cells at 0.5 mA cm⁻^2^ current density using different electrolytes. Scanning electron micrographs (SEM) of electrodes after 4 mAh cm⁻^2^ stripping in b) LNBG, c) LNG, and d) LTG electrolytes. SEM of electrodes after 1 mAh cm⁻^2^ plating in e) LNBG, f) LNG, and g) LTG electrolytes. SEM of electrodes after 4 mAh cm⁻^2^ plating in h) LNBG, i) LNG, and j) LTG electrolytes.

Symmetric Li|Li cells were utilized to perform galvanostatic measurements, employing three distinct electrolytes: 1.0 M lithium nitrate (LiNO₃) + 0.05 M lithium bromide (LiBr) in tetraethylene glycol dimethyl ether (G4), denoted as LNBG; 1.0 M LiNO₃ in G4, denoted as LNG; and 1.0 M lithium bis(trifluoromethanesulfonyl)imide (LiTFSI) in G4, denoted as LTG. These electrolytes are widely adopted in Li−Air and Li−S batteries, where the Li metal electrode must endure high‐capacity cycling.^[^ ^25^, ^26^, ^27^, ^28^, ^29^ ^]^ The addition of LiBr in such systems is a well‐known approach to lower charge overpotential in Li−Air batteries and result in a polished Li metal surface.^[^ ^30^, ^31^, ^32^, ^33^ ^]^ The cells were cycled at a constant current density of 0.5 mA cm⁻^2^ with an areal capacity of 4 mAh cm⁻^2^. **Figure** **1**a displays the voltage profiles for the first Li stripping and plating cycles for each electrolyte. The LTG electrolyte exhibited the lowest voltage polarization, which can be attributed to its superior ionic conductivity, as demonstrated in Figure S1 (Supporting Information). Notably, despite having comparable ionic conductivities, LNG and LNBG showed slight differences in voltage polarization, suggesting differences in the morphological evolution of Li in these two systems.^[^ ^34^ ^]^ The surface morphologies of the Li electrodes following 4 mAh cm⁻^2^ of stripping in each electrolyte were characterized by SEM. As shown in Figure 1b, the LNBG electrolyte produced a smooth and uniform Li surface, indicative of homogeneous stripping behavior. In contrast, the LNG electrolyte led to significant surface inhomogeneity, with irregular regions extending throughout the electrode, as observed in Figure 1c and the magnified SEM image in Figure S2 (Supporting Information). The most pronounced physical damage to the electrode surface was observed in the LTG electrolyte, where stripping resulted in extensive surface roughness and pit formation (Figure 1d).

The morphologies of Li deposits with an areal capacity of 1 mAh cm⁻^2^ formed on previously stripped electrodes were characterized using SEM, as depicted in Figure 1e–g. The SEM analysis revealed distinct variations in Li deposition behavior across the three electrolyte systems. In the LNBG electrolyte, Li deposition produced a smooth, compact layer with a uniform surface texture, indicative of continuous and homogeneous growth. This flat morphology in Figure 1e suggests that Li plated uniformly across the electrode surface without preferential nucleation sites or significant structural irregularities. In contrast, the LNG electrolyte facilitated a significantly different deposition pattern. As shown in Figure 1f, Li deposits in this system exhibited a spherical morphology, with discrete nucleation sites distributed across the electrode surface. This pattern implies a progressive nucleation‐and‐growth mechanism, where Li preferentially nucleates at localized regions of higher conductivity and charge density. The resulting spherical particles had diameters ranging from 5 to 30 µm. As the deposition continued, once the electrode surface was completely covered with a layer of spherical particles, further nucleation and growth occurred vertically, forming subsequent layers of Li spheres. This stack‐by‐stack deposition mechanism resulted in a 3D, non‐compact structure. A more strikingly different morphology emerged in the LTG electrolyte, where Li deposition led to the formation of whisker‐like structures. As illustrated in Figure 1g, these loosely entangled Li whiskers displayed highly anisotropic growth, with long, thread‐like deposits emerging from spatially isolated nucleation sites. Similar to the LNG electrolyte, nucleation was localized, but in this case, the subsequent growth proceeded in a highly anisotropic manner. To investigate the temporal evolution of these Li deposition morphologies, SEM observations were repeated after increasing the deposition capacity to 4 mAh cm⁻^2^. As shown in Figure 1h, the LNBG electrolyte maintained its layer‐by‐layer deposition behavior, forming a dense and compact Li layer with no significant changes to the surface morphology over time. This consistency indicates that the deposition process remained homogeneous, without the formation of isolated nucleation sites. For the LNG electrolyte, shown in Figure 1i, the overall bulk morphology remained unchanged during prolonged deposition. The primary difference observed was a more complete surface coverage by the Li spheres, as the deposition progressed. Rather than any noticeable transition to a different growth mode, the LNG electrolyte continued to promote progressive nucleation and growth, resulting in a thicker deposit with multi‐stacked particle‐like Li. A similar temporal trend was observed for the LTG electrolyte. As observed in Figure 1j, the whisker‐like deposition pattern persisted with increasing deposition capacity, with Li continuing to grow randomly and upwards, forming an increasingly thick and porous structure. Notably, no discernible transition to a more compact morphology was observed, even as more Li accumulated on the electrode surface.

To gain deeper insights into the temporal evolution of Li deposition, cross‐sectional SEM was performed after reaching a deposition capacity of 4 mAh cm⁻^2^. As shown in Figure S3a (Supporting Information), the deposition in the LNBG electrolyte exhibited a compact, layer‐by‐layer growth pattern. Energy‐dispersive X‐ray spectroscopy (EDS) mapping (Figure S3b–d, Supporting Information) of the cross‐section indicated that the SEI layer remained intact at the top of the electrode. This observation suggests that Li deposition occurred beneath the SEI layer. An EDS line scan near the electrode surface (Figure S4a–d, Supporting Information) estimated the SEI thickness to be ≈0.75 µm. In contrast, the Li deposition morphology in the LNG electrolyte, as revealed through cross‐sectional SEM (Figures S5, S6, Supporting Information), displayed a distinct multi‐stacked structure composed of spherical Li particles. The SEM image in Figure S5a (Supporting Information) shows that the deposited layer had a thickness of ≈30 µm, which is 10 µm thicker than the theoretical thickness (20 µm) of a monolithic Li layer expected for a capacity of 4 mAh cm⁻^2^. This thickness deviation suggests a non‐compact deposit with interparticle voids contributing to the increased electrode volume. The corresponding EDS mapping (Figure S5b–d, Supporting Information) demonstrated that oxygen and carbon were distributed throughout this 30 µm layer, indicating the presence of SEI layers in the interstitial space of the spherical Li particles. A more detailed view of the cross‐section near the surface (Figure S6a, Supporting Information) revealed roughly three distinct layers of these stacked spherical Li particles. EDS line scan results (Figure S6b–d, Supporting Information) confirmed that each individual Li sphere was coated with an SEI layer thicker than 1 µm. A markedly different deposition behavior was observed in the LTG electrolyte. As shown in the cross‐sectional SEM image in Figure S7a (Supporting Information), Li deposition in this electrolyte produced a significantly thicker (∼ 60 µm) and more porous layer than that observed in the other electrolytes. The EDS mapping (Figure S7b–d, Supporting Information) confirmed the presence of oxygen and carbon throughout the deposition layer, suggesting that the whisker‐like Li structures were also covered with an SEI layer. A higher‐magnification SEM image (Figure S8a, Supporting Information) and EDS line scans (Figure S8b–d, Supporting Information) provided clear evidence of a relatively thick SEI coating the Li whiskers, contributing to the porous, non‐compact morphology. Notably, no changes in the bulk morphology, thickening of the individual whisker, or densification of the overall deposits were observed during the progress of deposition.

**Figure 2:**
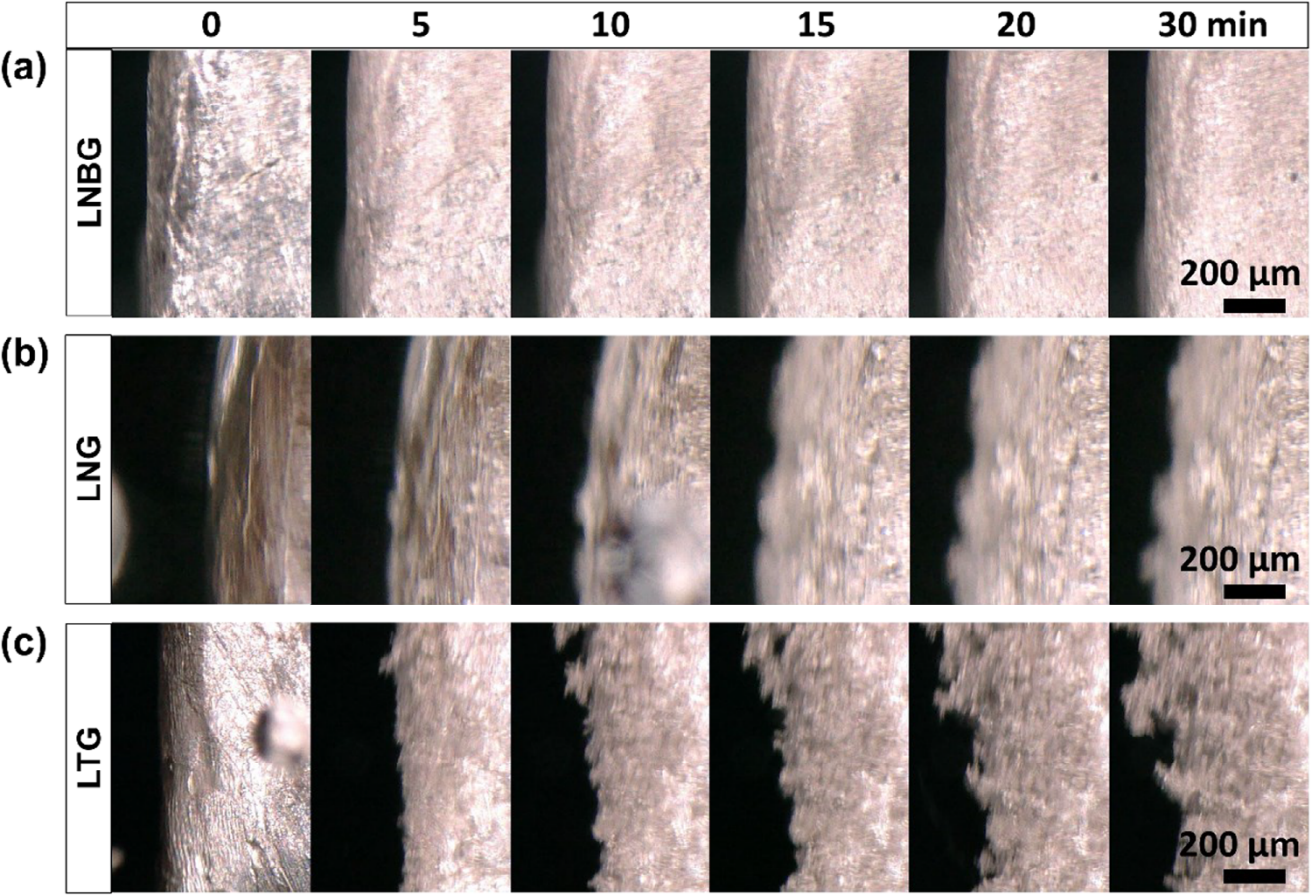
In situ optical micrographs of Li deposition on Li electrodes at selected time intervals in a) LNBG, b) LNG, and c) LTG electrolytes.

The variations in nucleation and growth patterns of Li in different electrolytes were further directly observed in situ using an OM for better temporal resolution. A schematic of the custom‐designed cell used for these real‐time optical observations is provided in Figure S9 (Supporting Information). Sequential snapshots captured at defined intervals during Li plating on a previously stripped Li electrode are presented in **Figure** **2**a–c, revealing significant differences in the deposition behavior among the three electrolyte systems. In the LNBG electrolyte, the deposition process exhibited remarkable homogeneity from the onset. As shown in Figure 2a, the electrode surface retained a smooth and consistent morphology throughout the plating process, with no observable changes in texture or evidence of localized nucleation. This indicates that Li deposition proceeded in a spatially uniform manner, with continuous growth occurring both laterally across the surface and vertically through successive layers. The absence of preferential deposition sites suggests a homogeneous flux of Li⁺ ions through the SEI, facilitating even surface coverage and suppressing the formation of isolated protrusions or 3D structures. By contrast, the LNG electrolyte demonstrated a more inhomogeneous nucleation pattern. As shown in Figure 2b, the initial stages of Li deposition were characterized by spatially isolated nucleation events. This lateral inhomogeneity persisted throughout the growth phase. The most pronounced anisotropic growth behavior was observed in the LTG electrolyte, where Li deposition produced whisker‐like structures. As depicted in Figure 2c, the formation of these elongated, porous Li whiskers originated from specific nucleation points, with growth proceeding outward and upward from the electrode surface. Although the precise nucleation site of individual whiskers was not resolvable at the magnification used, the overall assessment confirms that Li deposition in LTG electrolytes is driven by highly uneven nucleation, leading to the development of a thick and porous network of Li filaments. Taken together, these real‐time OM observations provide compelling evidence that the nucleation behavior, growth patterns, and resulting Li morphologies are strongly electrolyte‐dependent. The deposition differences are apparent from the very first stages of nucleation, with LNBG supporting continuous, homogeneous layer formation, LNG promoting discrete spherical particle growth, and LTG facilitating anisotropic, whisker‐like structures. The effects of morphological differences among the three electrolytes are also evident in their cycling stability. Figure S10 in Supporting Information presents the cycling performance and Coulombic efficiency (CE) of Li|Cu cells cycled at 0.5 mA cm⁻^2^ with a capacity of 4 mAh cm⁻^2^ in LNBG, LNG, and LTG electrolytes. While the cell cycle in LTG failed due to a short circuit within 50 h (Figure S10a, Supporting Information), the cells cycled in LNBG (Figure S10b, Supporting Information) and LNG (Figure S10c, Supporting Information) remained stable for over 300 h. The average CE values (Figure S10d, Supporting Information) during the first three cycles also showed significant differences, exceeding 90% for LNBG and LNG, but remaining ≈30% for LTG.

### 2.2 The Microstructure of Electrodeposited Li in Different Electrolytes

**Figure 3:**
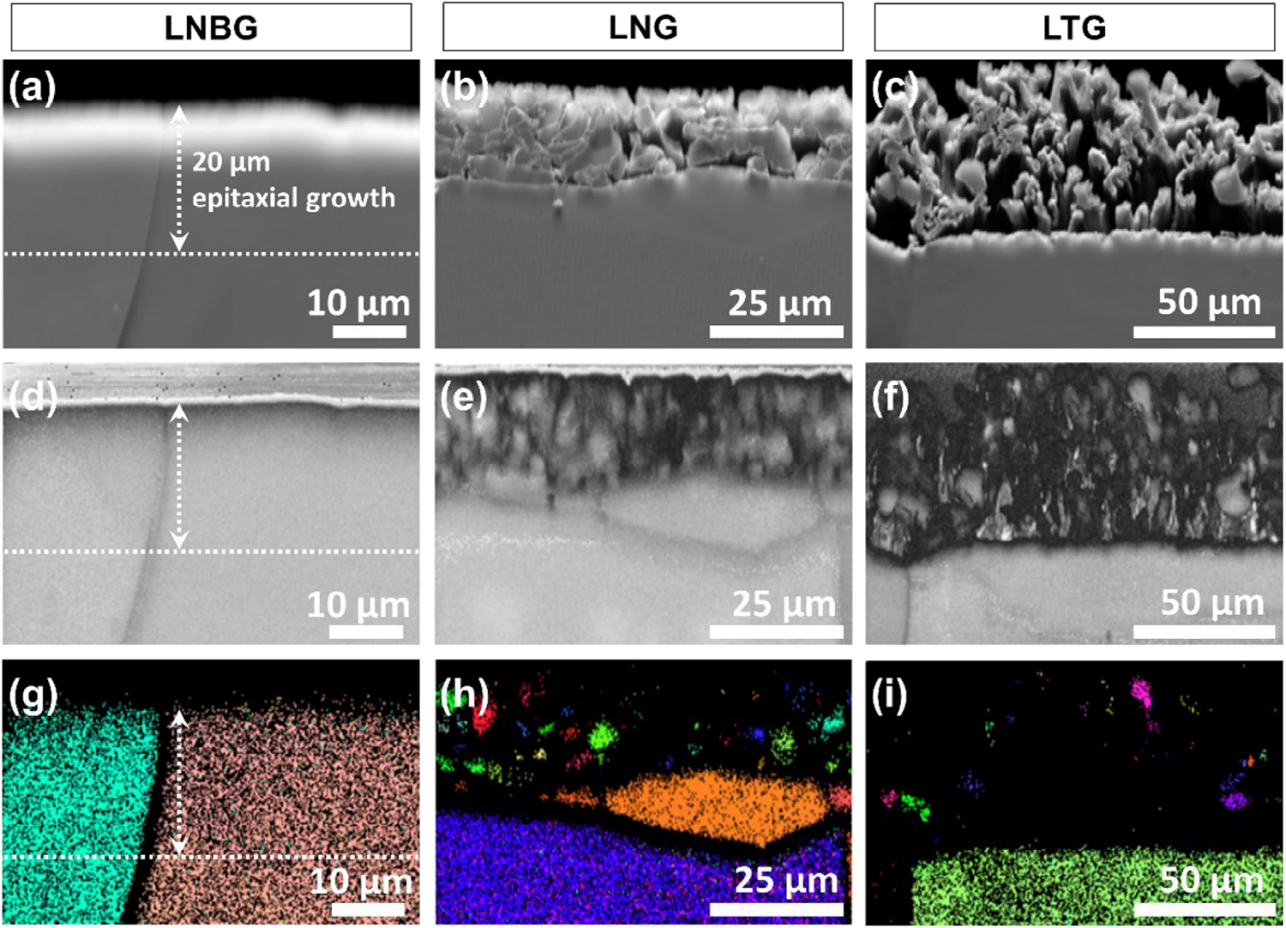
a–c) Forescatter detector (FSD) images, d–f) band contrast (BC) images, and g–i) inverse pole figure (IPF) maps of the Li electrode cross‐sections after 4 mAh cm⁻^2^ plating in LNBG, LNG, and LTG electrolytes respectively.

EBSD is a powerful technique for characterizing the microstructure, grain size, and crystallographic orientation of materials.^[^ ^16^, ^17^ ^]^ To investigate these characteristics in the deposited Li, EBSD analysis was performed on cross‐sections of electrodes following Li deposition with a capacity of 4 mAh cm⁻^2^ in the three different electrolytes. Before the measurements, the electrode cross‐sections were polished using argon (Ar) ion milling to ensure a smooth surface suitable for high‐resolution EBSD imaging. **Figure** **3**a–c presents forescatter detector (FSD) images of the electrodes after Li deposition in LNBG, LNG, and LTG electrolytes, respectively. The compact (Figure 3a), spherical (Figure 3b), and loosely entangled whisker‐like (Figure 3c) morphologies observed are consistent with the SEM results, confirming the distinctive deposition patterns for each electrolyte. To further examine the crystallographic quality of the deposits, band contrast (BC) images of the same cross‐sections are shown in Figure 3d–f. In these images, brighter regions indicate areas with well‐defined crystallographic orientations and high‐quality diffraction patterns, whereas darker regions suggest grain boundaries, high‐strain zones, or areas with poor pattern quality. The BC image of the electrode deposited in the LNBG electrolyte (Figure 3d) reveals a relatively uniform distribution of bright regions throughout the cross‐section, including the deposited Li layer, except for darker regions near the top surface and along a vertical line in the center, which likely correspond to the SEI layer and a grain boundary, respectively. In contrast, the BC images for LNG and LTG electrolytes (Figure 3e–f) show that only the unplated portion of the electrode at the bottom exhibits consistent brightness, while the deposited regions contain alternating bright and dark areas. The appearance of the dark areas can be attributed to the presence of SEI layers surrounding each deposited particle, inter‐particle voids, and difficulties in achieving the optimal 70° tilt alignment relative to the electron beam during EBSD mapping of the loosely connected, porous Li deposits. The inverse pole figure (IPF) maps of the deposited Li electrodes along the *x* direction are displayed in Figure 3g–i, where each distinct color corresponds to an individual Li crystal grain. The IPF map for the LNBG electrolyte (Figure 3g) reveals that the deposited Li maintained a consistent crystallographic orientation with the underlying electrode, indicating epitaxial, layer‐by‐layer growth. This observation is corroborated by the IPF images from the *y* and *z* directions (Figure S11a,b Supporting Information), which further confirm that grain alignment is preserved across all spatial dimensions. The deposition patterns in LNBG were verified by repeat EBSD imaging, shown in Figure S12 (Supporting Information). The continuous and homogeneous flux of Li ions during deposition likely plays a central role in enabling this conformal crystallographic matching, resulting in a dense, uniform Li layer. In contrast, the IPF map for the LNG electrolyte (Figure 3h) reveals a markedly different crystallographic relationship. Unlike the LNBG case, the deposited Li grains in the LNG electrolyte exhibit no clear orientation correlation with the underlying electrode structure. Interestingly, however, each individual spherical Li particle within the deposit appears to grow as a single crystal, despite the absence of a preferred crystallographic facet for nucleation. This observation is further validated by the IPF images from the *y* and *z* directions (Figure S13a,b Supporting Information), which confirm that these spherical particles consist of single grains. This suggests that, while the initial nucleation sites are random and uncorrelated with the lattice structure of the underlying electrode, the subsequent growth of each Li particle proceeds uniformly, forming isolated single‐crystalline domains. For the LTG electrolyte, IPF mapping proved particularly challenging due to the highly porous and whisker‐like nature of the Li deposits. Nevertheless, certain regions in Figure 3i and the supplementary images (Figure S14a,b Supporting Information) reveal elongated, anisotropic Li whiskers composed of single grains extending up to 10–15 µm in length. This clear evidence of single‐crystalline growth in LTG electrolytes aligns with prior findings obtained via cryogenic transmission electron microscopy (cryo‐TEM).^[^ ^35^ ^]^ Notably, these whiskers grew with random crystallographic orientations, suggesting that while the nucleation process in this electrolyte does not support grain matching with the underlying substrate, once initiated, the whisker‐like structures maintain their single‐grain integrity throughout the growth process.

### 2.3 The Origin of Electrolyte‐Dependent Microstructural Differences in Electrodeposited Li

A critical question that emerges from these observations is: What governs the differences in Li nucleation and growth patterns across these electrolytes? To understand the origin of the distinct nucleation and growth patterns of Li and the resulting microstructure and bulk morphologies observed in different electrolytes, it is essential to consider the interfacial processes governing Li deposition. During Li plating in the presence of an SEI, solvated Li⁺ ions must transport through the bulk electrolyte to the interface, undergo desolvation, cross the SEI layer, and finally reduce at the electrode surface.^[^ ^18^, ^21^ ^]^ Thus, the transference number of Li⁺ ions (*t₊*), charge‐transfer kinetics at the electrolyte‐SEI interface, the diffusion of Li⁺ ions through the SEI, and the spatial uniformity of this transport play pivotal roles in determining the deposition behavior.^[^ ^18^, ^36^ ^]^ In this context, the Li⁺ transference numbers (*t₊*) of LiNO₃‐based electrolytes are higher than that of the LiTFSI‐based system. The *t₊* values for LNG and LNBG are reported to be 0.51 and 0.56, respectively, whereas LTG exhibits a lower value of 0.41.^[^ ^18^ ^]^ Another critical factor influencing these patterns is the local current density distribution during deposition. Low charge‐transfer resistance often leads to uneven current density, with preferential deposition occurring at regions of higher local current, which in turn promotes sporadic nucleation and the formation of protrusions.^[^ ^18^ ^]^ According to established phase‐field models for metal deposition, faster interfacial kinetics exacerbate this effect by generating higher current densities at the tips of growing deposits, thereby facilitating anisotropic, tip‐driven growth.^[^ ^37^ ^]^ In contrast, higher charge‐transfer resistance helps to mitigate this uneven current distribution, supporting more uniform nucleation and layer‐by‐layer deposition of Li.^[^ ^18^, ^38^ ^]^ To evaluate the role of electrolyte‐specific kinetics, we measured the impedance of symmetric Li|Li cells after three stripping/plating cycles (4 mAh cm⁻^2^) in each electrolyte system. As shown in Figure S15a,b (Supporting Information), both LNBG and LNG electrolytes exhibited significantly higher charge‐transfer resistances (104 and 87 Ω, respectively) compared to the LTG electrolyte (64 Ω). Notably, the ionic resistance through the SEI layer was lower in both LiNO₃‐containing electrolytes (LNBG and LNG) (Figure S15c, Supporting Information). The layer‐by‐layer, compact Li growth with crystallographic continuity observed in LNBG electrolytes can be attributed to the synergistic effect of favorable charge‐transfer kinetics and a highly uniform interphase. The latter likely results from the electropolishing action of LiBr, which creates a smooth electrode surface and ensures a consistent flux of Li⁺ ions.^[^ ^31^ ^]^ In the LNG electrolyte, despite the similarly high charge‐transfer resistance, localized areas on the SEI surface (as shown in Figure 1c; Figure S2, Supporting Information), characterized by enhanced conductivity, charge density concentration, or mechanical imperfections such as cracks, act as spatially isolated nucleation sites for Li deposition. These inhomogeneities lead to sporadic nucleation at specific locations, preventing lattice matching with the substrate and resulting in the random crystallographic orientations observed in the isolated Li particles. As Li particles grow at these sites, they become encapsulated by a fresh SEI layer, which forms concurrently with the deposition process. As the SEI layer reaches a critical thickness, ion diffusion becomes restricted, limiting particle growth and prompting the nucleation of additional Li spheres on the surface of previously deposited structures. The formation of a thick (>1 µm) SEI layer around each growing particle prevents the coalescence of adjacent grains, maintaining the single‐crystalline yet spatially disconnected nature of the deposits. The slower deposition kinetics in this system favor the growth of discrete, spherical Li particles rather than anisotropic structures. Similarly, the LTG electrolyte, with its highly heterogeneous interphase, promotes nucleation at specific localized sites. However, unlike LNBG and LNG electrolytes, the faster kinetics in LTG electrolytes result in preferential deposition at the tips of growing Li structures, triggering self‐amplifying anisotropic growth and the formation of whisker‐like morphologies.^[^ ^36^ ^]^ Despite this directional growth, the whiskers themselves retain their single‐grain nature, highlighting the fact that crystallographic continuity within each Li structure can persist even under conditions of rapid and uneven deposition. These findings highlight the crucial role of electrolyte‐dependent deposition kinetics and interphasial properties in governing Li nucleation and growth. Homogeneous interphase combined with slower kinetics facilitates uniform, layer‐by‐layer deposition, whereas heterogeneous interfaces and faster kinetics promote spatially localized, anisotropic growth. In LNG and LTG electrolytes, where interphasial inhomogeneity is present from the initial stripping stage, Li deposition maintains consistent nucleation and growth patterns at higher capacities, with no significant changes in morphology or crystallographic orientation. This raises an important question: does the LNBG electrolyte maintain epitaxial Li growth as the deposited layer thickens, or does the system eventually transition to a different growth mode? In the context of thin‐film epitaxy, it is well known that beyond a critical thickness, strain accumulation at the interface often induces a transition from 2D layer‐by‐layer growth to 3D island formation, a phenomenon described by the Stranski–Krastanov (S–K) growth model.^[^ ^12^ ^]^ Investigating whether such a transition occurs during Li deposition in LNBG electrolytes is crucial for understanding its long‐term stability and performance in high‐capacity LMBs.

### 2.4 Temporal Evolution of Li Microstructure During Ultra‐High‐Capacity Deposition

**Figure 4:**
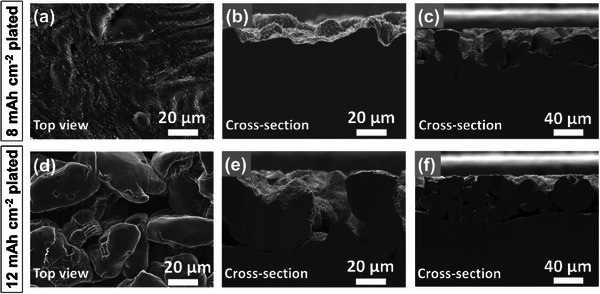
Top view and cross‐sectional SEM images of Li electrodes after a–c) 8 mAh cm⁻^2^ and d–f) 12 mAh cm⁻^2^ plating in LNBG electrolyte.

To further examine the morphological and crystallographic evolution of Li at ultra‐high capacities, we extended our SEM and EBSD analyses to investigate Li deposition in the LNBG electrolyte up to 12 mAh cm⁻^2^. **Figure** **4**a presents the top‐view SEM image of the electrode after a deposition capacity of 8 mAh cm⁻^2^. The surface appears largely compact, though small bumps and depressions begin to emerge, suggesting the onset of structural irregularities. Cross‐sectional SEM images in Figure 4b,c provide additional insight into this morphological transition. In the region shown in Figure 4b, the Li deposit continues to grow in a layer‐by‐layer manner, maintaining a predominantly planar morphology, although with increasing surface roughness. In contrast, Figure 4c reveals a different region of the same electrode, where the layered growth transitions into a particle‐like morphology after a certain deposition capacity. This observation suggests a non‐uniform deposition mechanism emerging at higher capacities. However, the SEM images alone cannot definitively determine whether these particle‐like features result from fresh, isolated nucleation events atop the 2D layered deposit or from strain‐induced cracking of the uneven portions of the layered structure. Regardless of the specific origin, it is evident that continuous 2D epitaxial growth breaks down at high capacity. As deposition continues, morphological instability sets in, driving a transition to a 3D growth mode. At even higher capacities, this transition becomes more pronounced. The top‐view and cross‐sectional SEM images of the electrode after deposition of 12 mAh cm⁻^2^, shown in Figure 4d‐f, reveal a complete shift to particle‐like growth. The surface is fully covered with spherical Li deposits, closely resembling the morphology observed in the LNG electrolyte at lower capacities.

**Figure 5:**
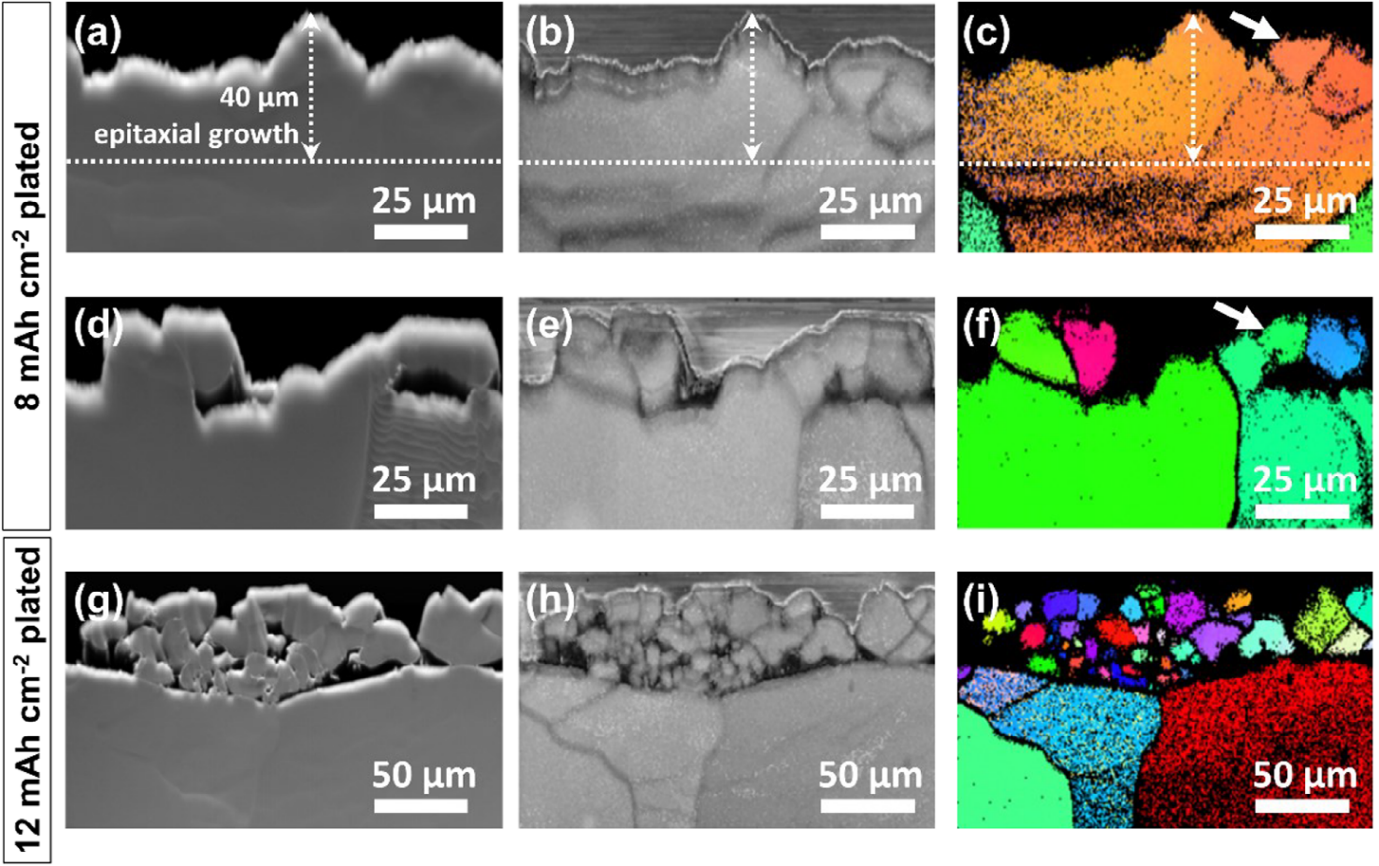
FSD, BC, and IPF images of the Li electrode cross‐sections after a–c) and d–f) 8 mAh cm⁻^2^, and g–i) 12 mAh cm⁻^2^ plating in LNBG electrolyte. a–c) and d–f) represent two different locations of the same Li electrode after 8 mAh cm⁻^2^ plating. White arrows in (c) and (f) show locations with subtle changes in color matching indicating changes in grain orientation, cracking, and the onset of particle formation.

**Figure** **5**a presents the FSD image of a cross‐sectional region of the electrode after 8 mAh cm⁻^2^ of deposition, revealing a compact Li layer with an uneven surface texture. This observation is consistent with the SEM image in Figure 4b, which suggested the onset of surface irregularities despite the continued layer‐by‐layer deposition pattern. A more detailed understanding emerges from the BC image in Figure 5b, where darker lines running through the seemingly compact deposit indicate the presence of grain boundaries or internal structural discontinuities. The IPF map along the *x* direction (Figure 5c) provides clear evidence of the crystallographic evolution. In addition to grains with distinctly different orientations, marked by contrasting colors and separated by grain boundaries, subtle changes in grain orientation become apparent at the growth front near the surface, as indicated by the arrow in the figure. These deviations indicated by the slightly different colors suggest the early stages of a breakdown in epitaxial growth. The IPF maps from the *y* and *z* directions (Figure S16a,b, Supporting Information) reinforce this observation, with Figure S16b (Supporting Information) particularly highlighting a clear deviation from crystal matching along the growth direction. To further investigate the transition from layered to particle‐like growth, we analyzed regions where visible particle formation had occurred atop the 2D Li layer. The FSD image in Figure 5d captures this morphological shift, showing the emergence of discrete Li particles interrupting the continuous film growth. The BC image in Figure 5e confirms the presence of both inter‐grain and inter‐particle separations, with dark lines outlining these structural boundaries. IPF maps of the same region from the *x*‐direction (Figure 5f) and the corresponding *y‐* and *z*‐direction maps (Figure S17a,b, Supporting Information) reveal a striking phenomenon: alongside deposits exhibiting subtle differences in crystal orientation, fresh Li particles with complete different crystallographic orientations nucleate as deposition proceeds. This observation suggests that even a minor mismatch in crystal orientation at the deposition front can trigger a complete breakdown of the epitaxial growth mode, forcing a transition to independent, randomly oriented nucleation events. After 12 mAh cm⁻^2^ of deposition, this transition becomes fully apparent. The FSD image in Figure 5g reveals a surface dominated by particle‐like Li deposits. The BC image in Figure 5h shows pronounced grain boundaries and inter‐particle SEI layers, confirming the structural discontinuities that accompany the morphological transition. Notably, the IPF maps (Figure 5i) and those in Figure S18a,b (Supporting Information) demonstrate that similar to the behavior observed in the LNG electrolyte, the spherical Li particles formed at this stage are predominantly single‐crystalline, having nucleated freshly atop the 2D layer. The microstructures of the deposited Li after 8 and 12 mAh cm⁻^2^ were verified by repeat EBSD imaging shown in Figures S19, S20 (Supporting Information). These results indicate that continuous 2D epitaxial growth in the LNBG electrolyte is fundamentally limited by crystal mismatches at high deposition capacity. During high‐capacity deposition, even slight deviations from crystallographic matching disrupt the epitaxial deposition pattern, initiating a transition to 3D nucleation and growth characterized by independent, single‐crystalline particles.

### 2.5 Temporal Evolution of Li Microstructure During Cycling

**Figure 6:**
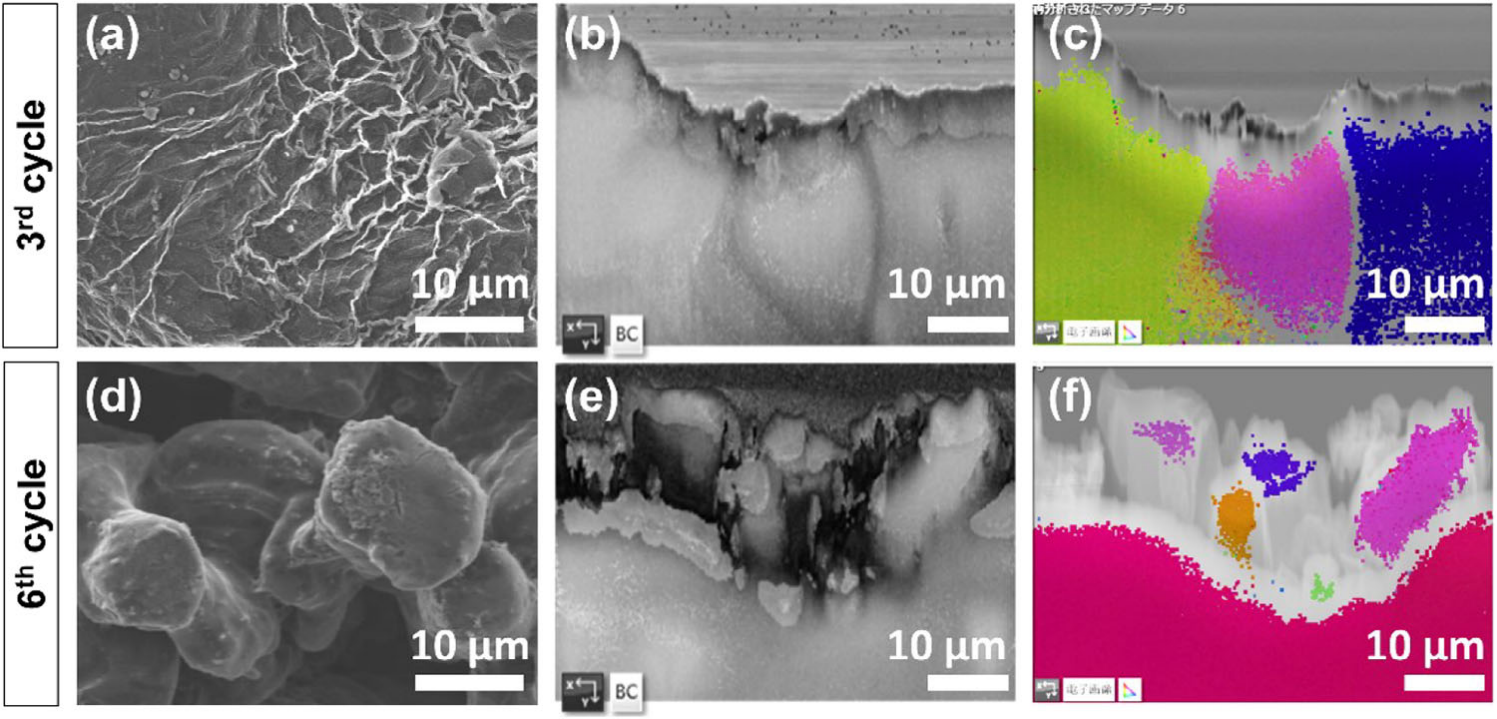
Top‐view SEM, BC, and EBSD layer images of the Li electrode cross‐sections after a–c) 3rd cycle and d–f) 6th cycle plating with a capacity of 4 mAh cm⁻^2^.

To further investigate the morphological and crystallographic evolution of Li deposition over multiple cycles, we performed SEM and EBSD analyses on electrodes after the third and sixth plating cycles, each with a deposition capacity of 4 mAh cm⁻^2^. The top‐view SEM image of the electrode after the third plating cycle (**Figure** **6**a) reveals a relatively compact Li layer with an increasingly rough surface. A more detailed perspective is provided by the cross‐sectional FSD image in Figure S21 (Supporting Information), which highlights variations in surface topography. The BC image in Figure 6b further illustrates the presence of grain boundaries and, notably, a distinct upper layer ≈5–8 µm thick, which appears structurally separate from the underlying Li layer. EBSD analysis with grain orientation mapping (Figure 6c) provides insight into the deposition process: the uppermost 5–8 µm does not exhibit grain mapping, suggesting the presence of a thick SEI layer, while the bottom part maintains an epitaxial growth. Importantly, no separate particle‐like grains are found to be formed. A more pronounced shift in deposition morphology is observed after the sixth plating cycle. The top‐view SEM image in Figure 6d reveals a fully developed particle‐like Li structure, in stark contrast to the earlier compact deposition. The corresponding cross‐sectional FSD image (Figure S22, Supporting Information) confirms that particle‐like growth dominates from the onset of deposition. The BC image in Figure 6e clearly delineates interparticle voids, further supporting a transition from layer‐by‐layer deposition to discrete particle formation. Additionally, EBSD analysis (Figure 6f) confirms that these newly formed particles exhibit single‐crystalline characteristics, indicating fresh nucleation events rather than continuous epitaxial growth. This transformation closely resembles the morphological evolution observed at an ultra‐high deposition capacity of 12 mAh cm⁻^2^. These findings indicate that, beyond a threshold of both deposition capacity and cycling, Li growth transitions from epitaxial layer‐by‐layer deposition to particle‐like nucleation. A key question that arises is the underlying mechanism driving this shift in crystallographic orientation during high‐capacity cycling.

### 2.6 Analysis of the Temporal Changes in the Interphasial Properties

**Figure 7:**
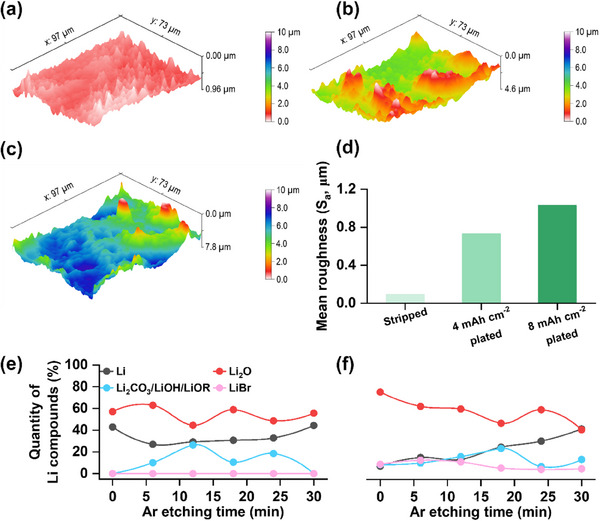
Surface topographic images of the Li electrodes after a) 12 mAh cm⁻^2^ stripping, b) 4 mAh cm⁻^2^ plating, and c) 8 mAh cm⁻^2^ plating in LNBG electrolyte. d) Mean surface roughness values of the electrodes quantified from the topography. Quantification of Li compounds by X‐ray photoelectron spectroscopy (XPS) at different depths of the electrode surface after (a) 12 mAh cm⁻^2^ stripping and f) 4 mAh cm⁻^2^ plating.

**Figure 8:**
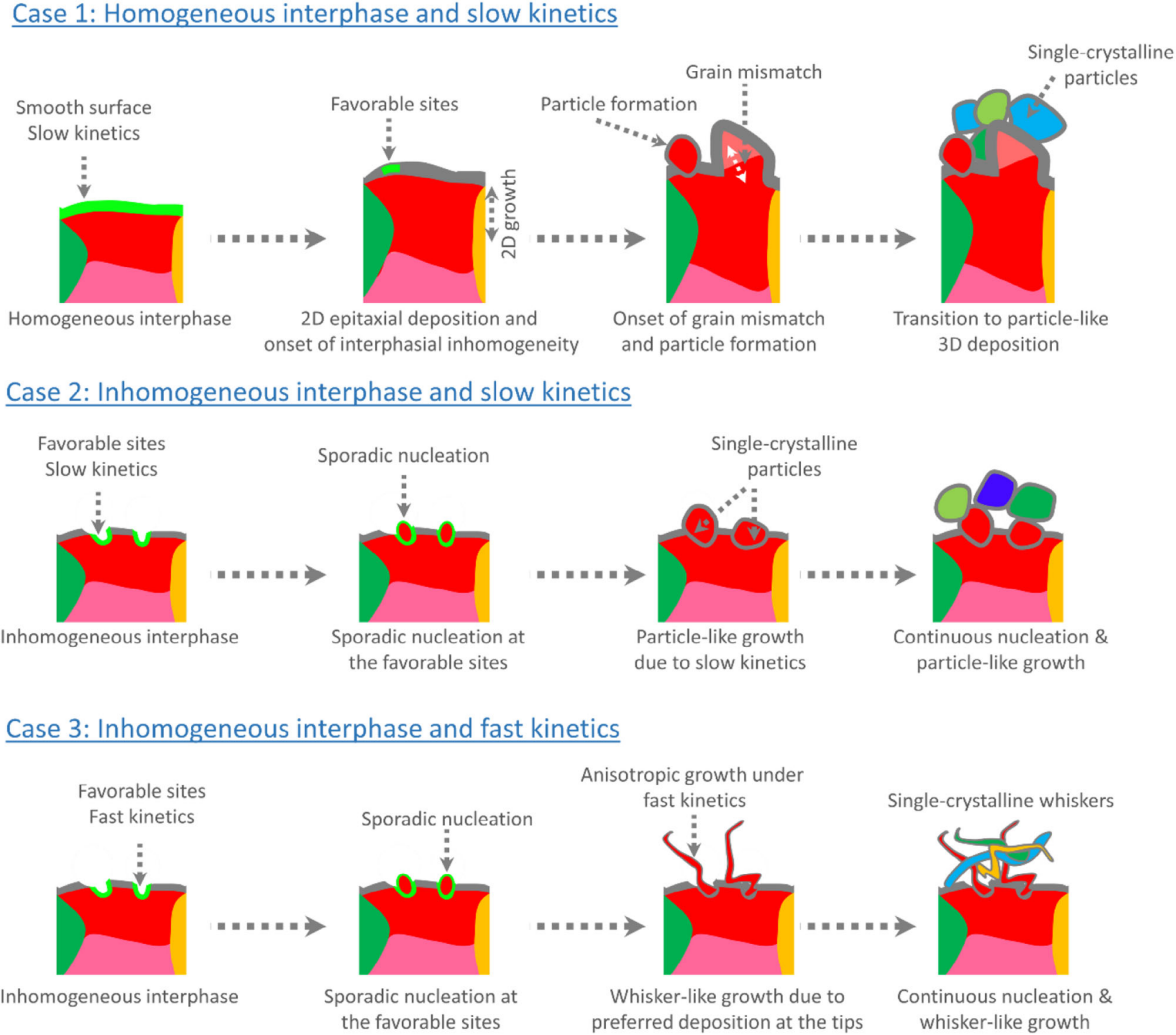
Schematic representation of temporal evolution of Li microstructures during plating in three different conditions. Each color represents a single grain of Li crystal.

Despite the similar ionic conductivity and deposition kinetics observed in LNBG and LNG electrolytes, the key factor differentiating their deposition morphologies appears to be their interphasial characteristics. This suggests that gradual changes in interphasial properties could also be responsible for the observed temporal evolution in Li deposition morphology within the LNBG electrolyte. A highly uniform flux of Li⁺ ions, combined with both lateral and vertical deposition uniformity at terrace sites, facilitates layer‐by‐layer epitaxial growth. However, any degradation in interphasial homogeneity can disrupt this stable growth process. The presence of surface protrusions can locally enhance charge density and promote preferential Li deposition due to the spherical diffusion flux of Li⁺ ions.^[^ ^39^, ^40^ ^]^ To quantitatively assess surface roughness variations during Li stripping and plating, we conducted 3D surface profiling using a laser microscope. The topographic image of the electrode after stripping for 12 mAh cm⁻^2^ (**Figure** **7**a) reveals a relatively smooth surface. In contrast, images captured after plating for 4 and 8 mAh cm⁻^2^ (Figure 7b,c) indicate a progressive increase in surface roughness. A quantitative analysis (Figure 7d) confirms this trend, with the mean surface roughness (S_a_) increasing from 0.093 µm after stripping to 0.73 µm following 4 mAh cm⁻^2^ deposition. At this stage, the epitaxial growth of Li is still preserved; however, the progressive increase in surface roughness indicates the onset of structural inhomogeneity, which has the potential to self‐amplify as deposition continues. Consequently, deposition to 8 mAh cm⁻^2^ results in even greater surface irregularity, with an S_a_ value reaching 1.03 µm. This phenomenon can be attributed to spatial variations in the properties of the SEI, leading to nonuniform Li deposition. To further investigate the role of SEI composition in these morphological changes, we conducted an XPS analysis of electrodes after 12 mAh cm⁻^2^ stripping and 4 mAh cm⁻^2^ plating (Figures S23, S24, Supporting Information). The quantification of the SEI compounds after stripping (Figure 7e) indicates that the SEI is primarily composed of lithium oxide (Li₂O), a component known to contribute to stable cycling performance in LMBs.^[^ ^30^, ^41^ ^]^ The detection of metallic Li both at the surface and after Ar etching suggests that the SEI remains relatively thin, enabling efficient Li⁺ transport. However, after 4 mAh cm⁻^2^ deposition (Figure 7f), the SEI still predominantly contains Li₂O, but the presence of metallic Li becomes less prominent near the surface and increases only with deeper etching, implying a thickening of the SEI. Gradual mechanical degradation of the interphase was also evident from the force‐displacement curves (Figure S25a,b, Supporting Information) obtained via atomic force microscopy (AFM) indentation measurements on the Li surface after 12 mAh cm⁻^2^ stripping followed by 4 mAh cm⁻^2^ plating. A comparison of the curves reveals that after the 4 mAh cm⁻^2^ plating step, the interphase exhibits increased inelastic behavior, as indicated by the pronounced hysteresis between the insertion and extraction curves. Additionally, the interphase becomes mechanically stiffer, and the presence of multiple inflection points in the insertion curve (Figure S25b, Supporting Information) suggests the formation of a multilayered interphase structure. These findings indicate that continued deposition leads to a loss of mechanical flexibility in the interphase. A similar investigation of interphasial degradation over multiple deposition‐stripping cycles was carried out by analyzing the electrodes after the third and sixth stripping cycles. The SEM images provided in Figures S26, S27 (Supporting Information) depict a progressive increase in surface roughness after the third and sixth stripping cycles, respectively. Quantification of the surface roughness using laser microscopy confirmed a clear trend of increasing surface irregularity with repeated cycling. Topographic images shown in Figures S28, S29 (Supporting Information), along with quantitative roughness data in Figure S30 (Supporting Information), further substantiate these findings. Specifically, the S_a_ of the electrode following the third stripping cycle is measured at 0.23 µm, while after the sixth stripping cycle, this value rises significantly to 0.65 µm. The XPS results of the electrode after the third strip in Figures S31, S32 (Supporting Information) further show an increase in chemical heterogeneity and also the thickness of the SEI layer during cycling. The progressive increase in surface roughness, along with the thickening and chemical inhomogeneity of the SEI layer, provides strong evidence that interphasial homogeneity deteriorates as both plating capacity and cycle number increase. The progressive interphasial inhomogeneity can be attributed to the continuous reaction between the electrolyte and the Li electrode, leading to the accumulation of electrolyte decomposition products. This degradation in interphasial properties appears to be a key factor driving the transition from epitaxial layer‐by‐layer Li deposition to 3D particle‐like growth during repeated plating cycles. As the SEI layer thickens, its ionic resistance increases, which in turn impedes the diffusion of Li⁺ ions across the interface. Variations in SEI thickness and composition across the electrode surface can disrupt the otherwise uniform flux of Li⁺ ions, leading to spatially heterogeneous deposition. These localized inconsistencies in ion transport create preferential deposition sites and such inhomogeneous deposition causes a shift in the Li nucleation and growth mechanism. Under homogeneous conditions, Li deposition occurs predominantly at terrace sites, enabling epitaxial layer‐by‐layer growth with minimal crystallographic mismatch. However, as interphasial uniformity deteriorates, the deposition process becomes increasingly influenced by the presence of surface protrusions and interphasial irregularities. The higher charge density around these protrusions, combined with the nonuniform diffusion of Li⁺ ions, facilitates deposition at kinetically preferred sites, rather than terraces. This deviation from layer‐by‐layer growth promotes irregular nucleation, which disrupts the crystallographic continuity of the growing Li structure, ultimately leading to a transition from epitaxial to 3D particle‐like deposition. **Figure** **8** provides a schematic representation of the microstructural evolution of Li as influenced by different kinetic and interphasial conditions discussed in this study.

## 3 Conclusion

In this study, we have systematically investigated the microstructural evolution of Li during ultra‐high‐capacity plating, focusing on the correlation between crystallographic orientation, deposition morphology, kinetic parameters, and interphasial properties in different electrolytes relevant to high energy density Li−Air and Li−S batteries. Using SEM, EBSD, XPS, and impedance analyses, we have demonstrated that both interphasial homogeneity and Li deposition kinetics play a dominant role in determining the crystallographic orientation and morphology of the deposited Li. Our findings reveal that a homogeneous SEI, combined with slow deposition kinetics, promotes layer‐by‐layer epitaxial Li growth with a coherent lattice orientation, ensuring a well‐ordered deposition process up to a certain capacity. However, as the deposition capacity increases or as cycling progresses, the SEI gradually degrades, leading to localized nucleation and growth of isolated Li particles that disrupt the epitaxial growth process. Even minor deviations in crystal lattice matching can trigger a complete breakdown of the initially stable deposition pattern, resulting in a transition to the nucleation and growth of particle‐like deposits with randomly oriented single‐crystalline grains. In contrast, when the SEI is inhomogeneous and the deposition kinetics are rapid, Li nucleation preferentially occurs at kinetically favorable sites. This leads to tip‐dominated growth and the formation of whisker‐like structures. These findings offer a generalized framework for understanding and controlling lithium microstructure formation, emphasizing the dual importance of interphasial homogeneity and deposition kinetics. To enable stable, epitaxial lithium deposition, future electrolyte and interface design must prioritize the formation of uniform SEI layers, whether through advanced liquid electrolytes, engineered artificial interphases, or solid‐state systems, alongside carefully tuned deposition kinetics. Looking ahead, further research is needed to quantitatively assess parameters such as interfacial roughness, mechanical strain, and stack pressure, all of which influence lithium growth at the microscale. While ex situ EBSD analysis has provided valuable insights, achieving a deeper understanding of interphase evolution and its interplay with electrochemical kinetics, particularly with improved temporal resolution through operando techniques, will be crucial for the rational design of next‐generation lithium metal electrodes and the advancement of safe, high‐energy‐density lithium metal batteries.

## Conflict of Interest

The authors declare no conflict of interest.

## Supporting information



Supporting Information

## Data Availability

The data that support the findings of this study are available from the corresponding author upon reasonable request.
