# Supplementary material for: Temporal Evolution of Lithium Metal Microstructures During Ultra‐High‐Capacity Stripping/Plating Cycles
Source: Adv Sci (Weinh). 2025 Jun 10;12(33):e06474. doi: 10.1002/advs.202506474 (PMC12412508; doi:10.1002/advs.202506474)
Supplement: Supplementary file 1 — Supporting Information [file ADVS-12-e06474-s001.docx]

Supporting Information

Temporal Evolution of Lithium Metal Microstructures During Ultra-High-Capacity Stripping/Plating Cycles

Arghya Dutta* and Yoshimi Kubo*

**1. Experimental section**

**1.1. Electrolyte preparation**

High-purity tetraethylene glycol dimethyl ether (tetraglyme or G4) with water content below 30 ppm was procured from Japan Advanced Chemicals and used as received, without further purification. The electrolyte salts, including lithium bis(trifluoromethanesulfonyl)imide (LiTFSI; KISHIDA CHEMICAL Co., Ltd.), anhydrous lithium nitrate (LiNO₃; KISHIDA CHEMICAL Co., Ltd.), and anhydrous lithium bromide (LiBr; Sigma-Aldrich), were subjected to a drying process at 120 °C under vacuum conditions for 24 hours within a super dry-room, maintaining a dew point below −60 °C. Electrolyte preparation was carried out entirely inside an argon (Ar) filled glovebox, where the dew point was maintained below −90 °C to minimize moisture contamination.

**1.2. Electrochemical cells**

To examine Li stripping and plating behavior, stacked Li|Li cells were utilized. Each stack was composed of two lithium metal foils (16 mm in diameter, 0.2 mm thick; sourced from Honjo Metal) with a Celgard® 2325 separator placed between them, which was saturated with 100 µL of electrolyte. All cell assemblies were performed within an argon-filled glovebox, ensuring a controlled environment with a dew point maintained below −90 °C. Electrochemical measurements were subsequently carried out under a continuous flow of oxygen (O₂) at approximately 20 mL min⁻¹, with the dew point also maintained below −90 °C.

For in situ optical microscopy observations of Li plating, a transparent glass tube cell (Figure S9) was employed. The spacing between the Li electrodes was fixed at 5 mm, and the electrodes were plated under a controlled current density of 0.5 mA cm⁻².

**1.3. Measurements and characterizations**

All galvanostatic Li stripping and plating experiments were conducted using a Hokuto Denko HJ1001SD8 electrochemical tester, while electrochemical impedance spectroscopy (EIS) measurements were performed with a Biologic VSP potentiostat/galvanostat. The ionic conductivities of the electrolytes were determined using a Mettler Toledo SevenExcellence conductivity meter.

Characterization of the Li electrodes, including surface morphology, chemical composition, and crystallographic orientation, was carried out using a JEOL JSM-7800F field emission scanning electron microscope (FE-SEM) equipped with an Oxford X-MaxN 50 energy-dispersive X-ray spectrometer (EDS) and an Oxford Nordlys Nano electron backscatter diffractometer (EBSD). In situ observations of Li stripping and plating were conducted using a Keyence VHX digital microscope. Three-dimensional (3D) surface profiling of electrode morphology was performed with a Keyence VK-X200 laser microscope. X-ray photoelectron spectroscopy (XPS) analysis was carried out using a ULVAC-PHY VersaProbe II Scanning XPS Microprobe, equipped with an argon gas cluster ion beam (Ar-GCIB) for surface cleaning and depth profiling. A Bruker AXS atomic force microscope (AFM) kept inside a glovebox was employed to measure the force-displacement curves.

All experimental procedures were conducted within an argon-filled glovebox (dew point maintained below −90 °C) or inside a super-dry room (dew point below −60 °C) to minimize exposure to moisture and atmospheric contaminants. For SEM and XPS measurements, hermetically sealed transfer vessels were used during sample transportation.

**Figure S1.** Ionic conductivities of different electrolytes measured at 25 °C.


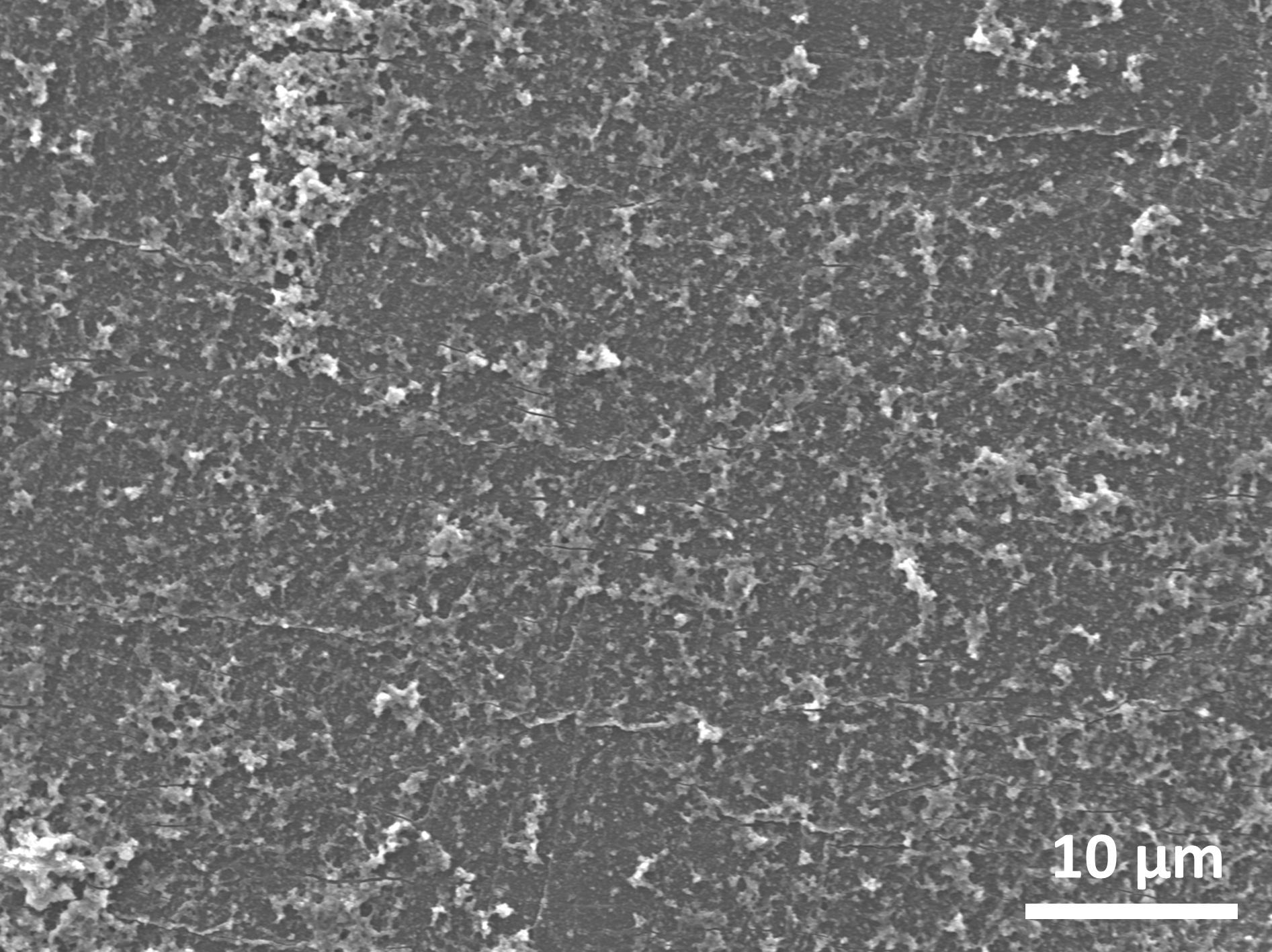


**Figure S2.** SEM image of the Li electrode stripped (4 mAh cm⁻²) in LNG electrolyte.


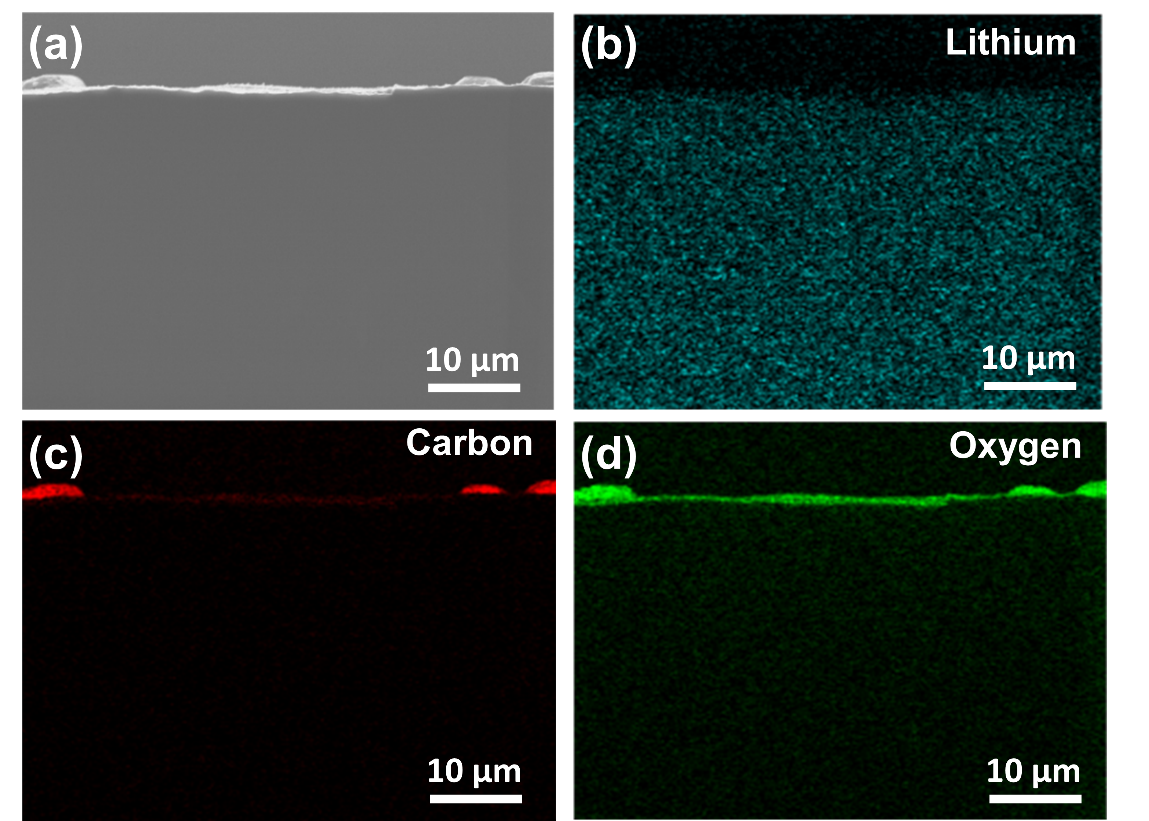


**Figure S3.** (a) Cross-sectional SEM and (b-d) EDS mapping images of the Li electrode plated (4 mAh cm⁻²) in LNBG electrolyte.


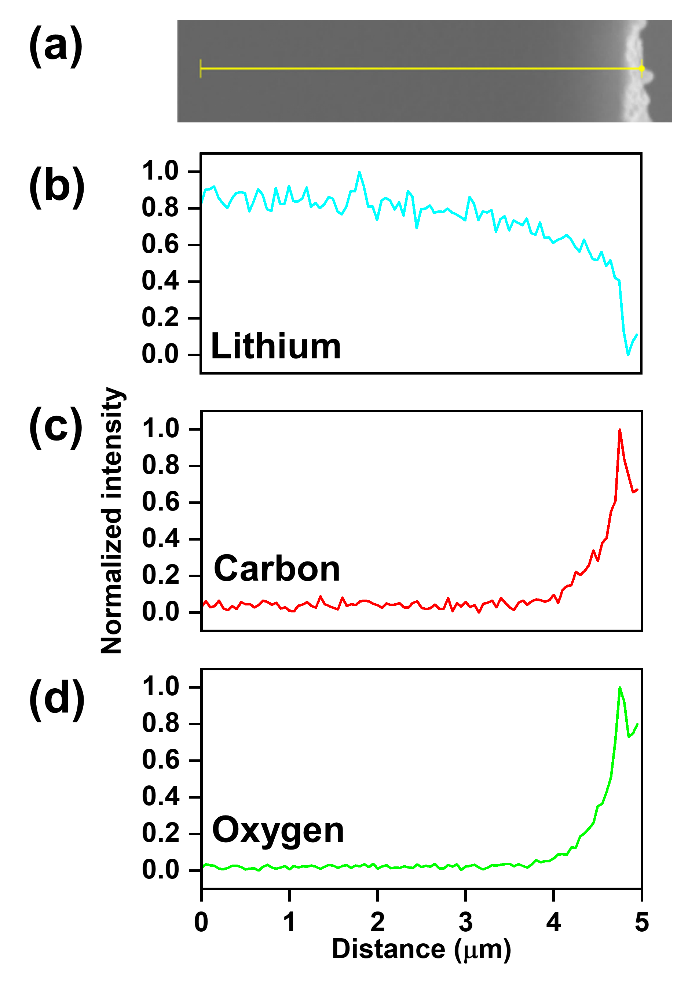


**Figure S4.** (a) Cross-sectional SEM and (b-d) EDS line scan images of the Li electrode plated (4 mAh cm⁻²) in LNBG electrolyte.


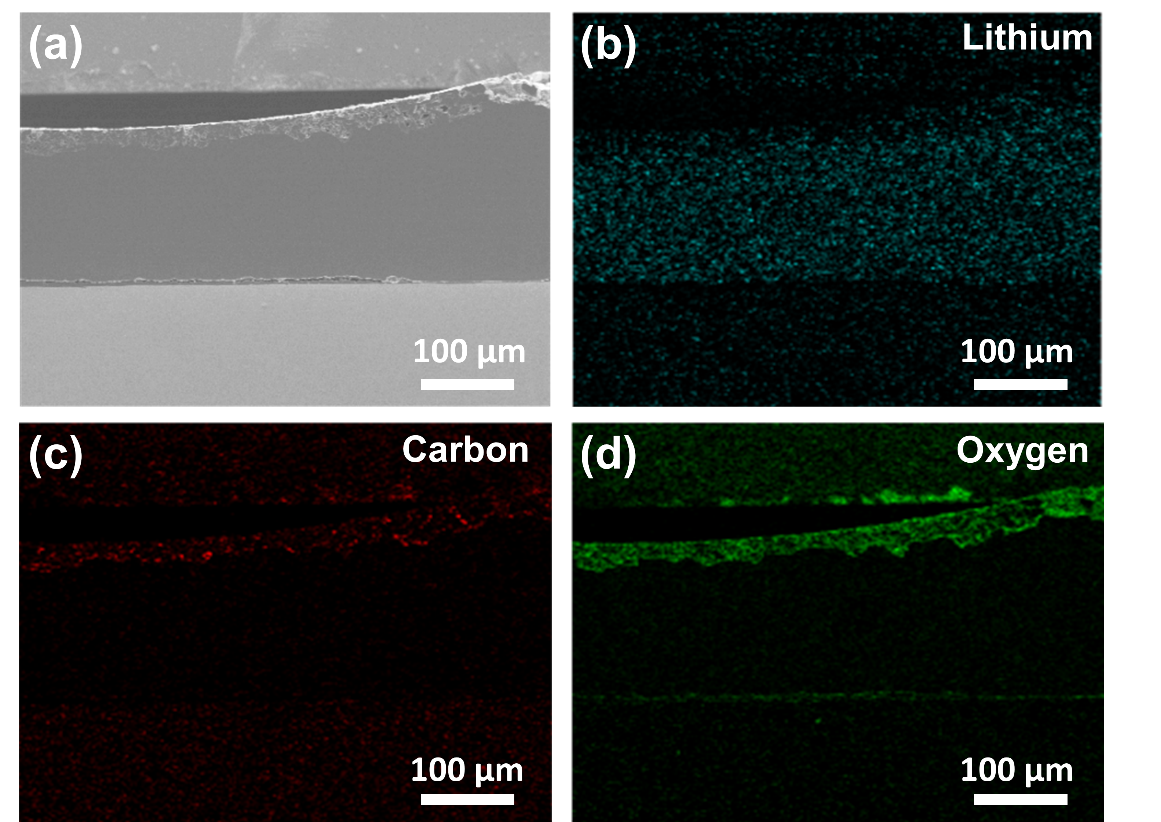


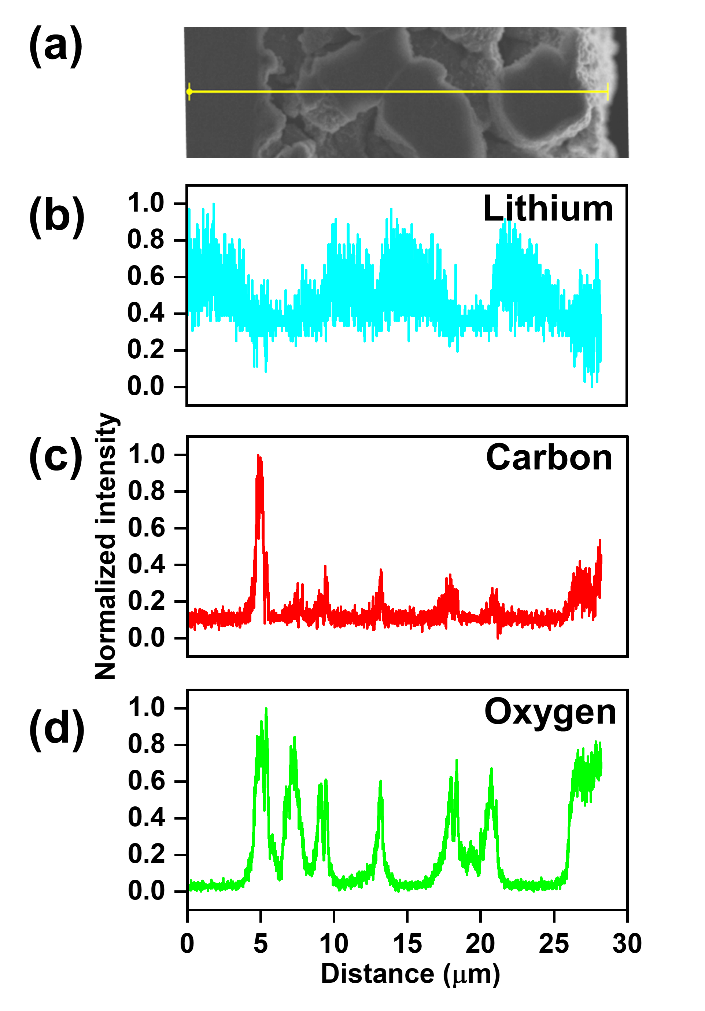
**Figure S5.** (a) Cross-sectional SEM and (b-d) EDS mapping images of the Li electrode plated (4 mAh cm⁻²) in LNG electrolyte.

**Figure S6.** (a) Cross-sectional SEM and (b-d) EDS line scan images of the Li electrode plated (4 mAh cm⁻²) in LNG electrolyte.


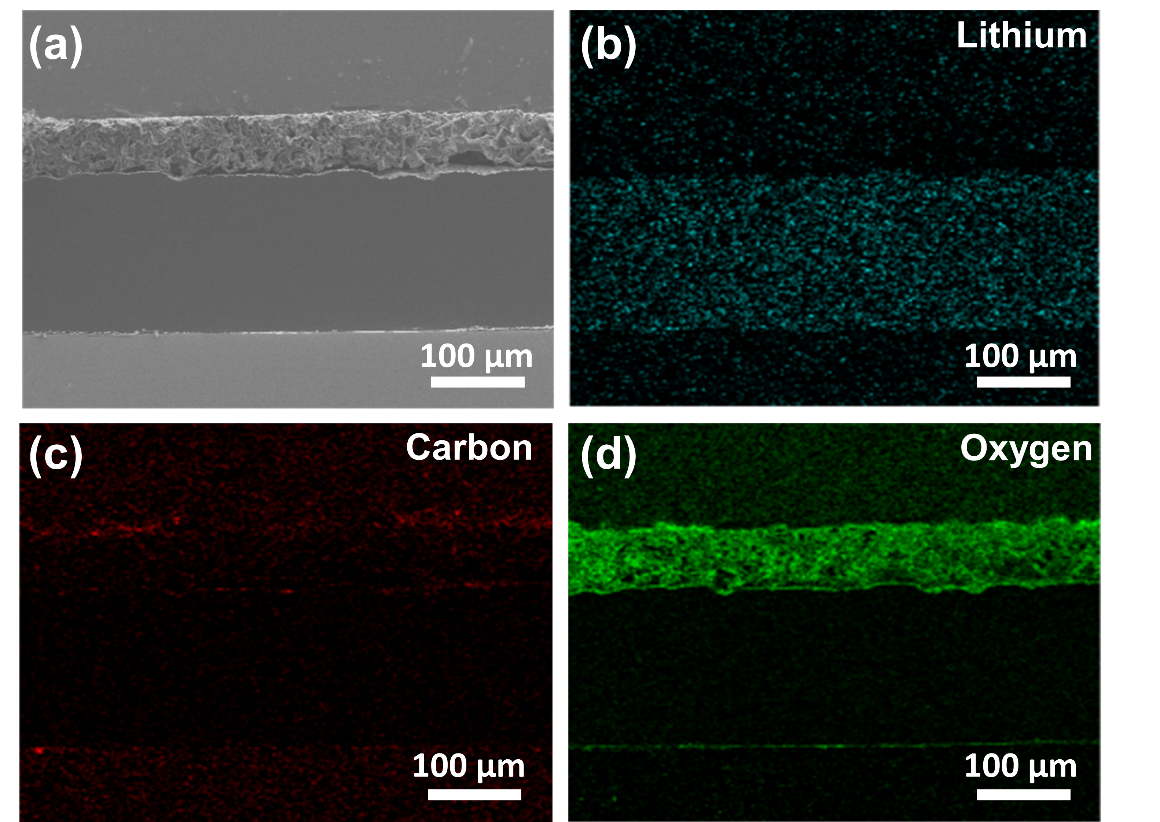


**Figure S7.** (a) Cross-sectional SEM and (b-d) EDS mapping images of the Li electrode plated (4 mAh cm⁻²) in LTG electrolyte.


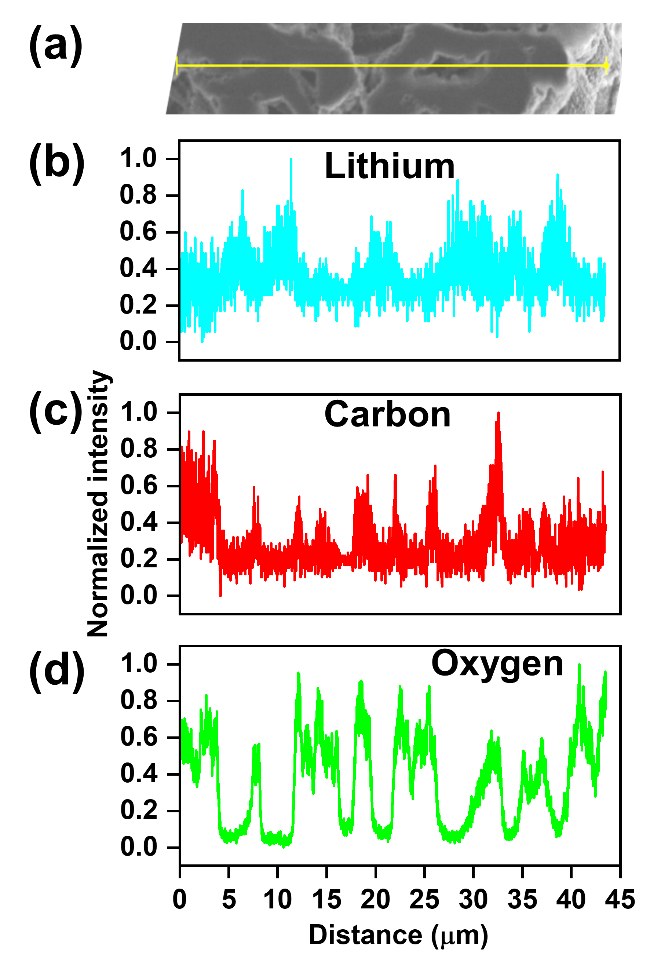


**Figure S8.** (a) Cross-sectional SEM and (b-d) EDS line scan images of the Li electrode plated (4 mAh cm⁻²) in LTG electrolyte.


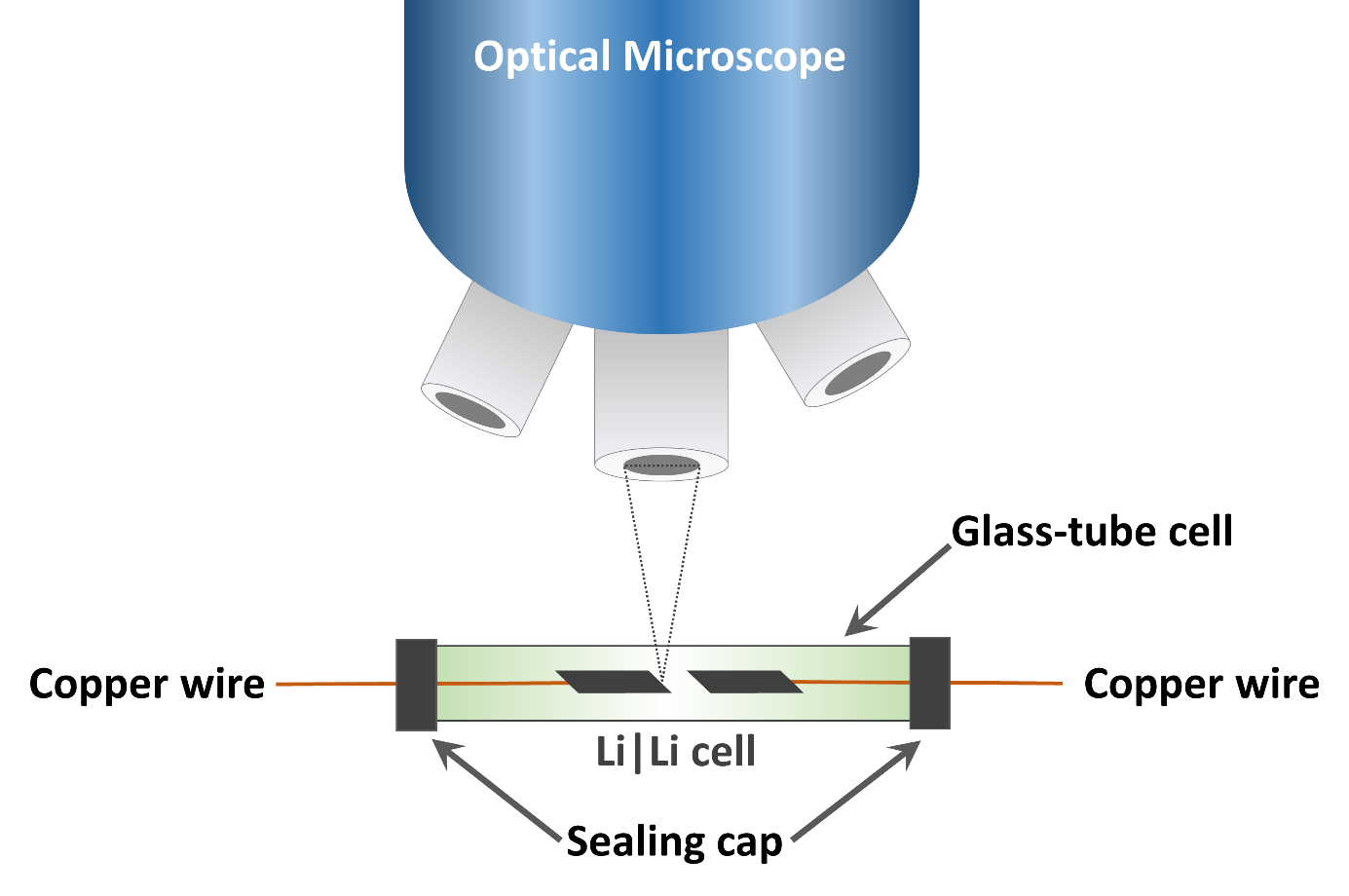


**Figure S9.** Schematic representation of the cell used for in situ optical microscopic experiment.


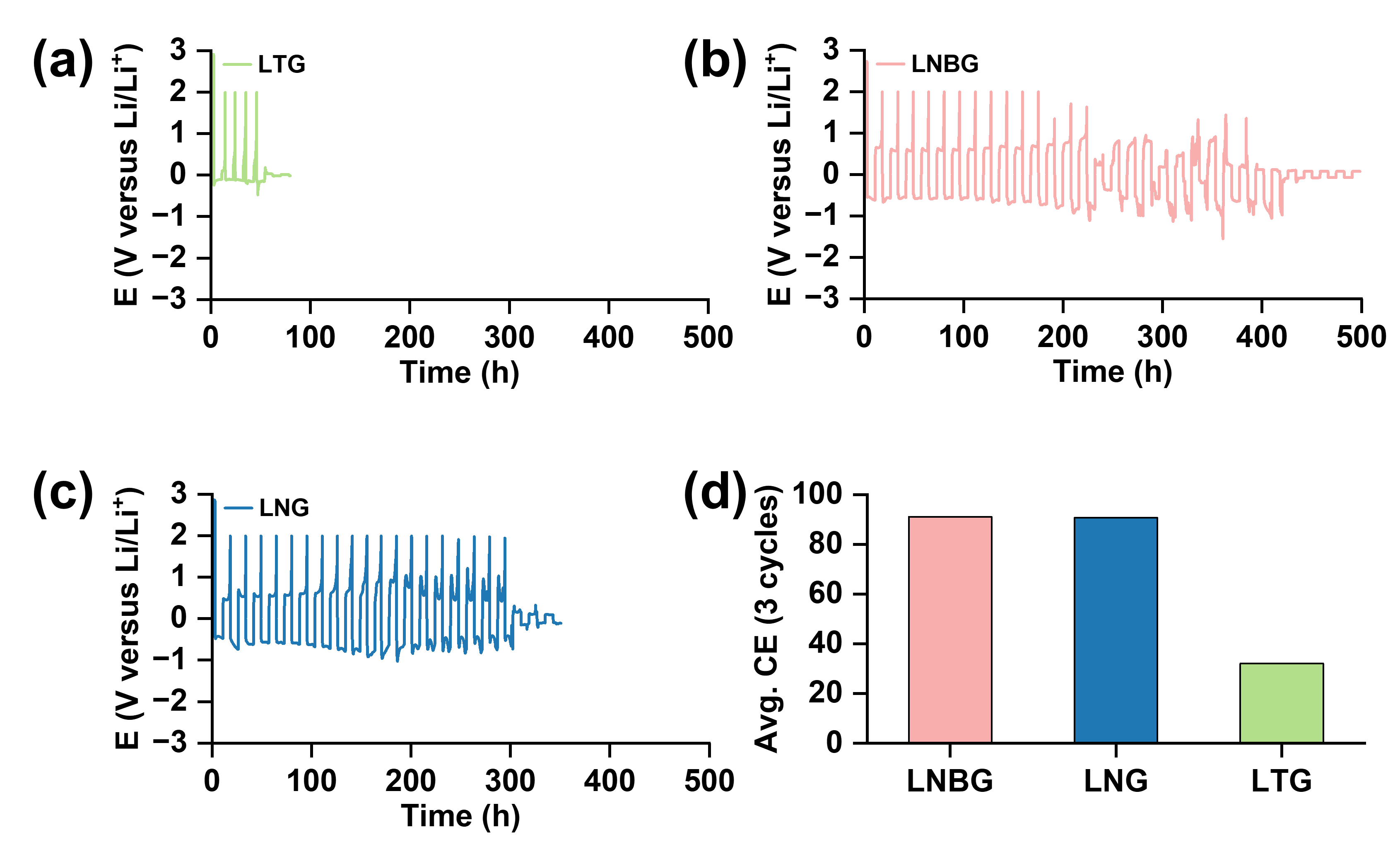


**Figure S10.** Galvanostatic clycling of Li|Cu cells in (a) LTG, (b) LNBG, and (c) LNG electrolytes at a current density of 0.5 mA cm⁻² and capacity of 4 mAh cm⁻². (d) Average Coulombic efficiency of the cells for the first 3 cycles.


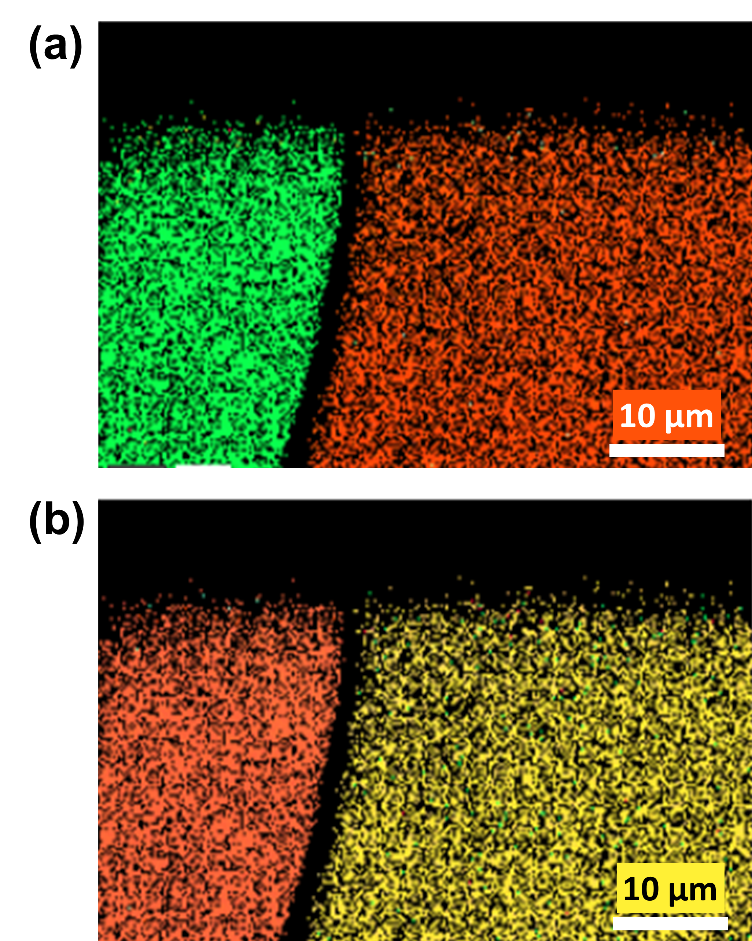


**Figure S11.** IPF mapping images of Li electrode cross-section after plating (4 mAh cm⁻²) in LNBG electrolyte from (a) *y* and (b) *z* directions.


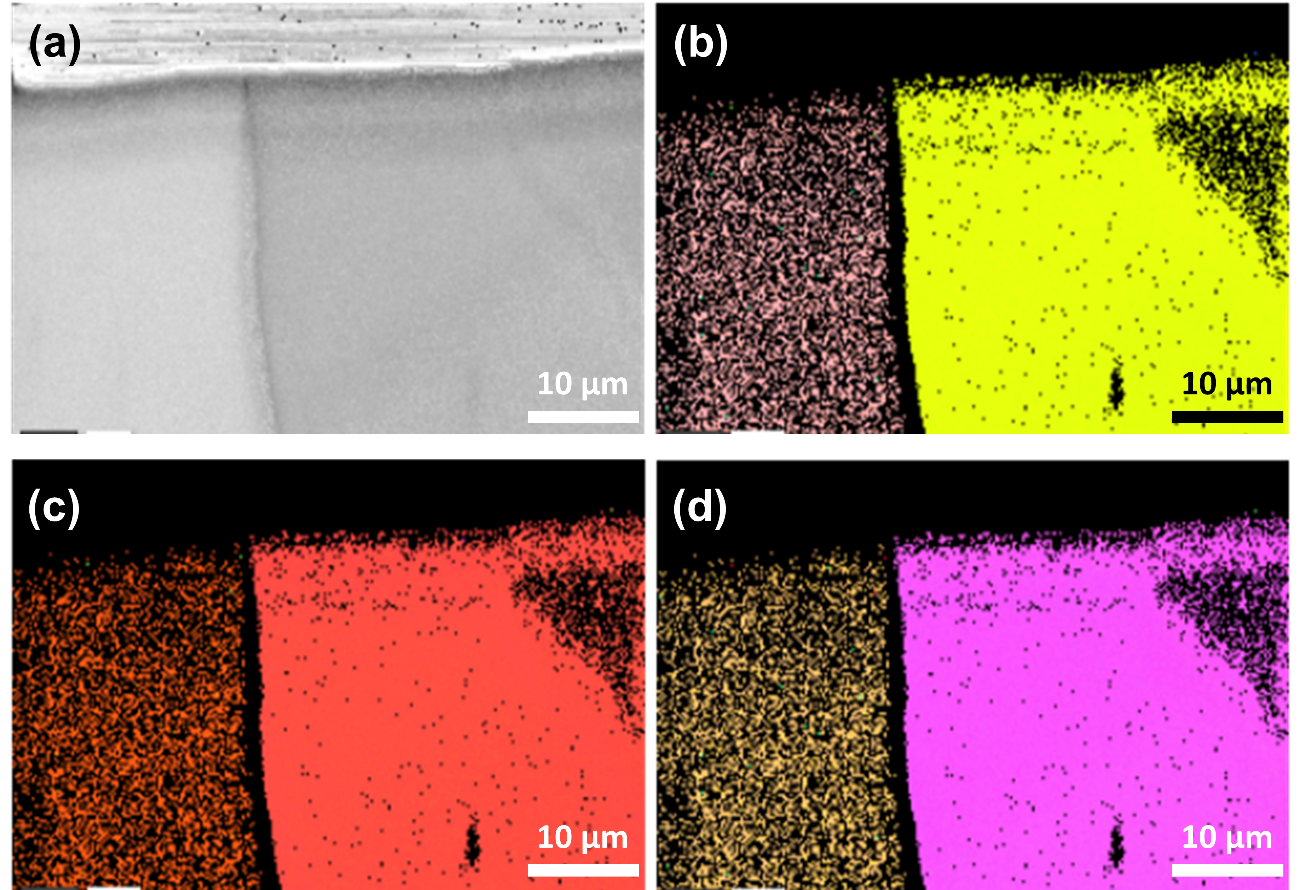


**Figure S12.** (a) BC and IPF mapping images from (b) *x*, (c) *y*, and (d) *z* directionsof Li electrode cross-section after plating (4 mAh cm⁻²) in LNBG electrolyte .


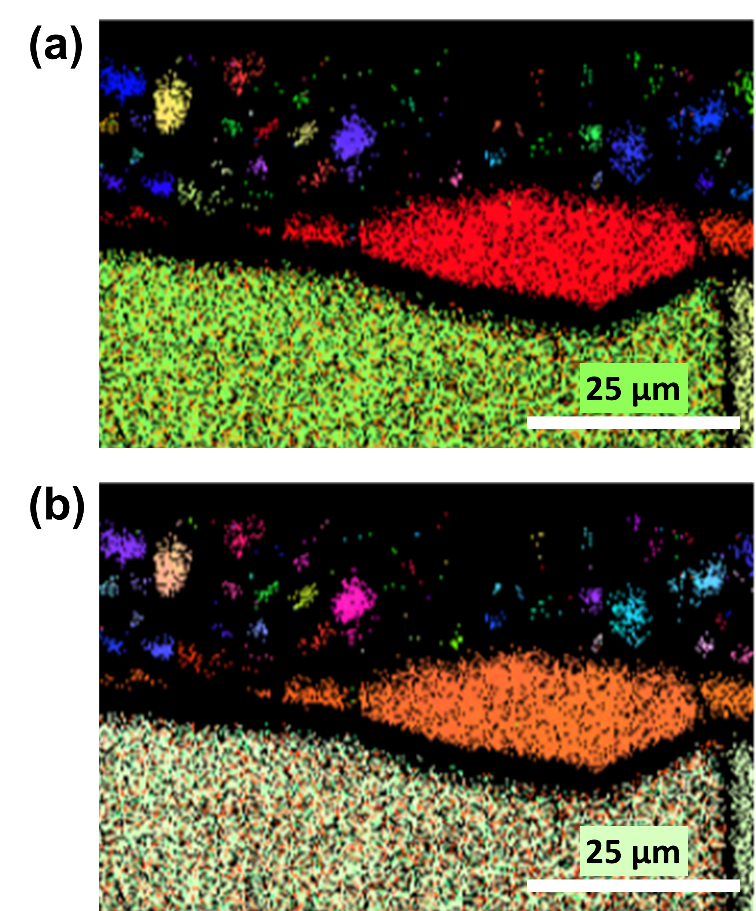


**Figure S13.** IPF mapping images of Li electrode cross-section after plating (4 mAh cm⁻²) in LNG electrolyte from (a) *y* and (b) *z* directions.


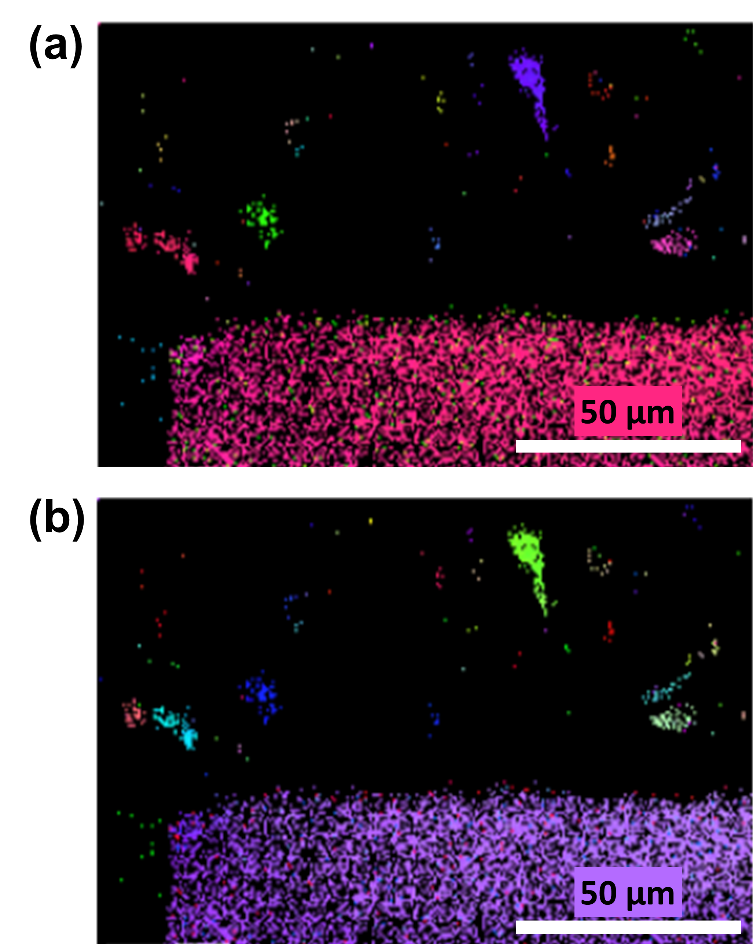


**Figure S14.** IPF mapping images of Li electrode cross-section after plating (4 mAh cm⁻²) in LTG electrolyte from (a) *y* and (b) *z* directions.


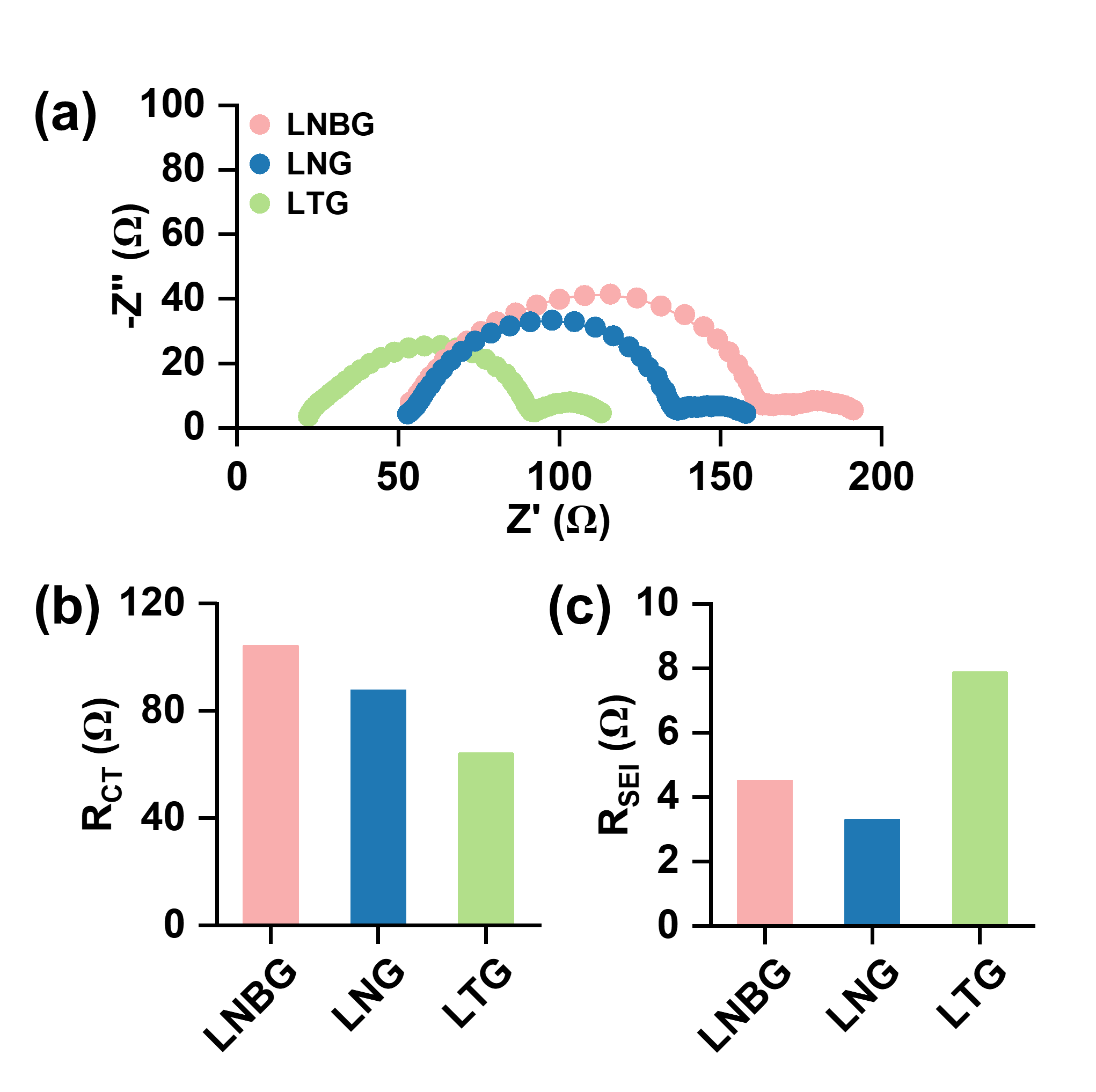


**Figure S15.** (a) Nyquist plot, (b) charge transfer resistance (R_CT_), and (c) interphasial resistance (R_SEI_) of Li|Li cells measured in different electrolytes.


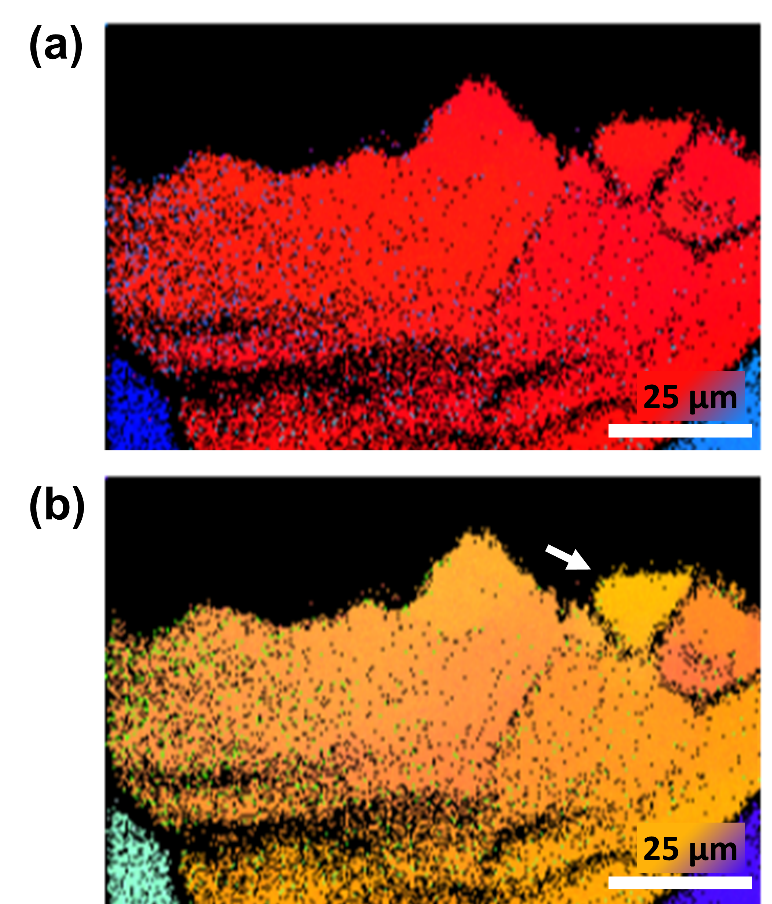
**Figure S16.** IPF mapping images of Li electrode cross-section after plating (8 mAh cm⁻²) in LNBG electrolyte from (a) *y* and (b) *z* directions.


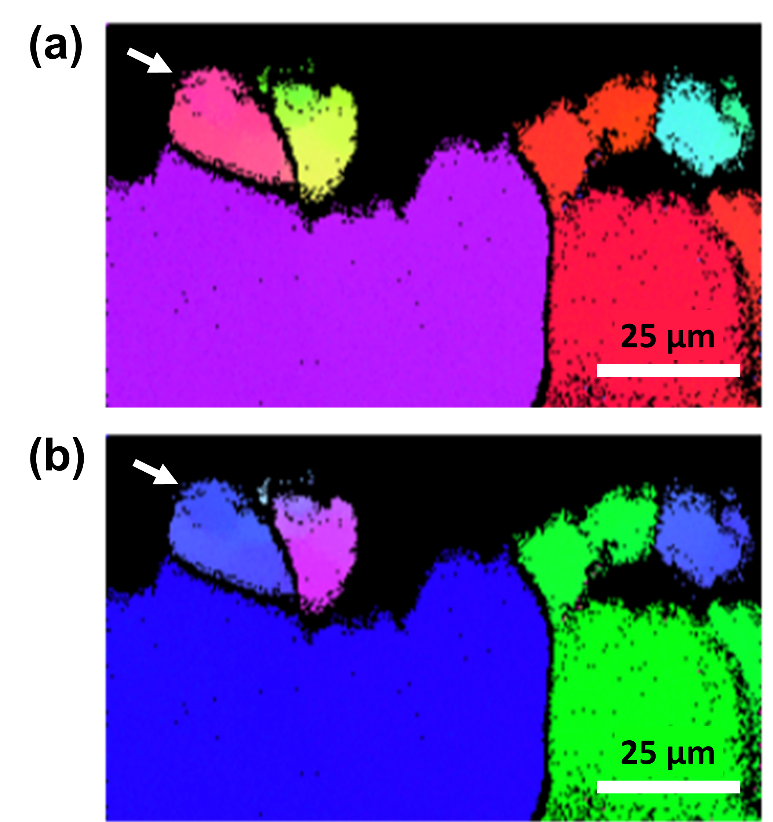


**Figure S17.** IPF mapping images of Li electrode cross-section after plating (8 mAh cm⁻²) in LNBG electrolyte from (a) *y* and (b) *z* directions. Locations of Figure S16 and Figure S17 are different in the same electrode.


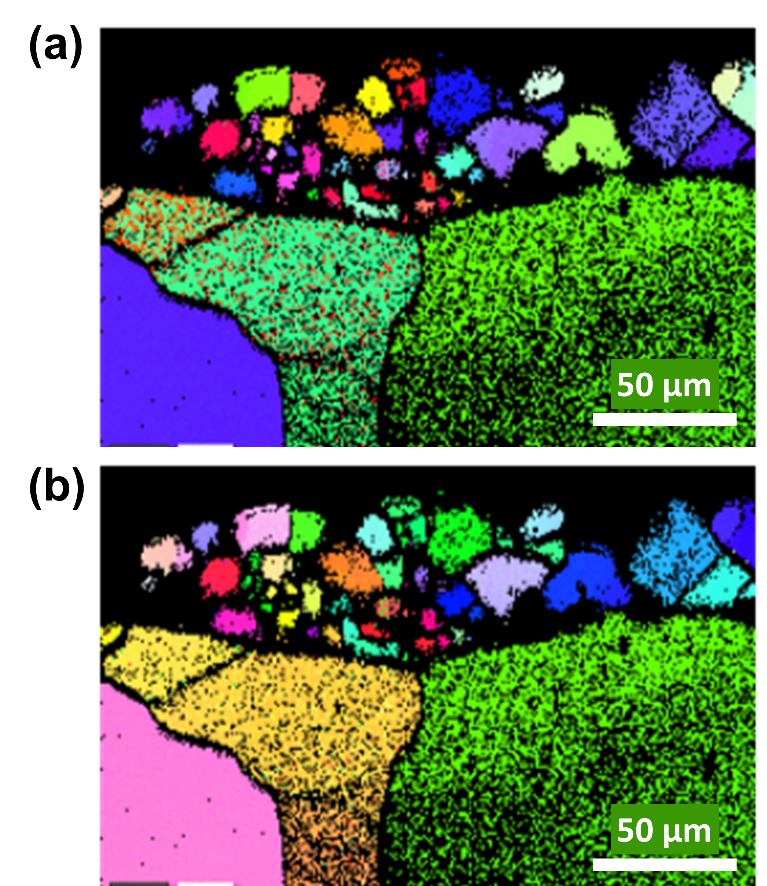


**Figure S18.** IPF mapping images of Li electrode cross-section after plating (12 mAh cm⁻²) in LNBG electrolyte from (a) *y* and (b) *z* directions.


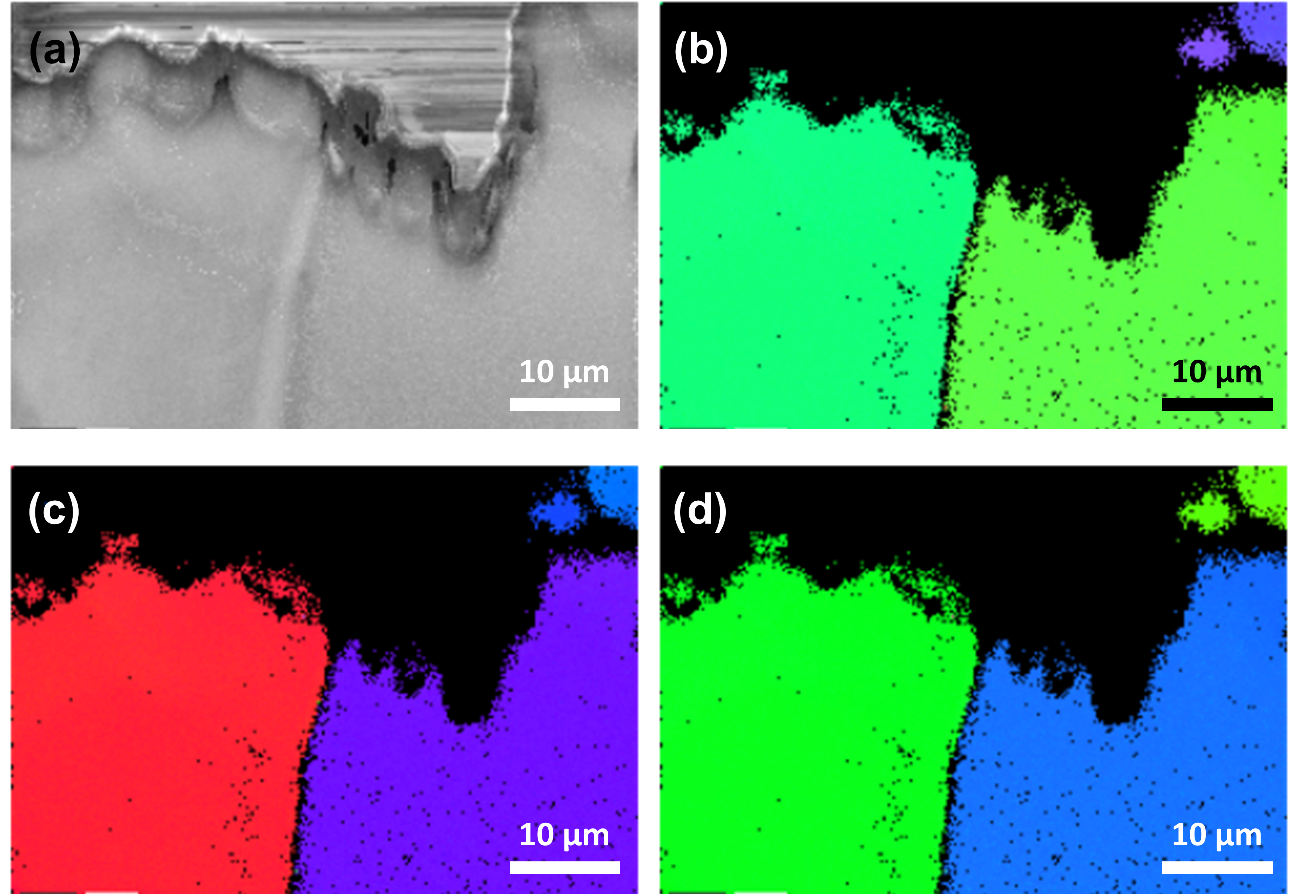


**Figure S19.** (a) BC and IPF mapping images from (b) *x*, (c) *y*, and (d) *z* directionsof Li electrode cross-section after plating (8 mAh cm⁻²) in LNBG electrolyte .


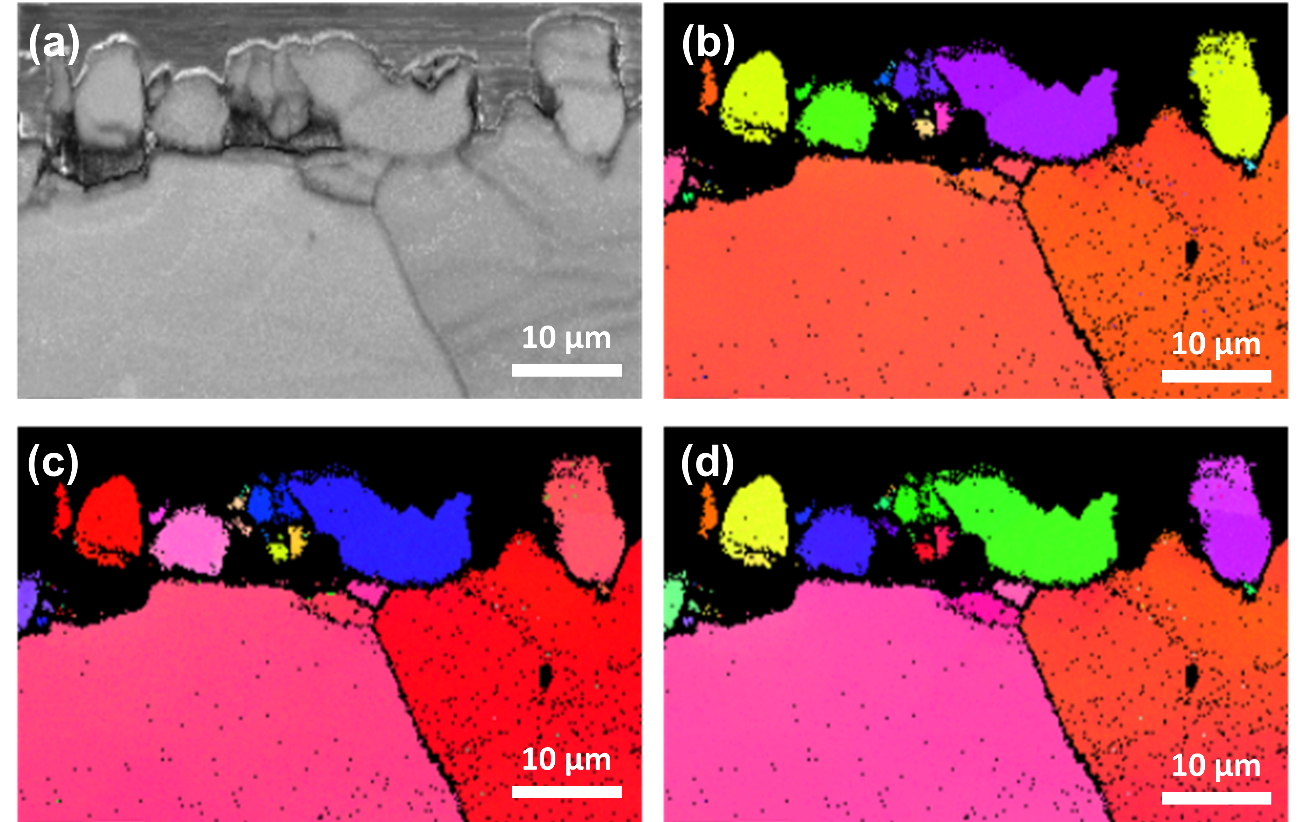


**Figure S20.** (a) BC and IPF mapping images from (b) *x*, (c) *y*, and (d) *z* directionsof Li electrode cross-section after plating (12 mAh cm⁻²) in LNBG electrolyte .


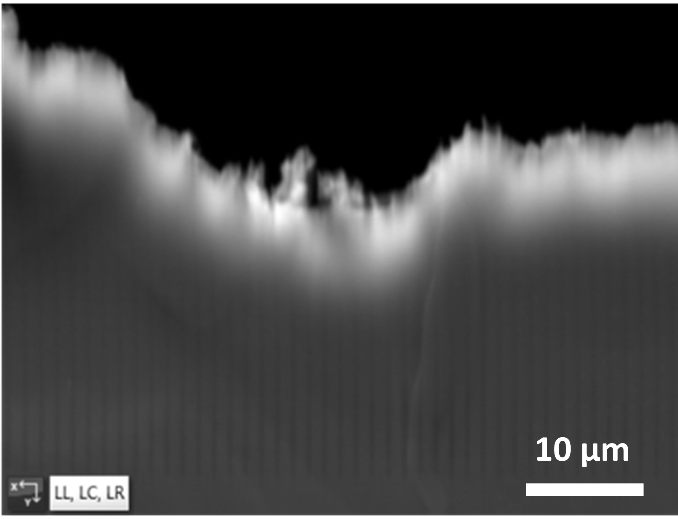


**Figure S21.** FSD image of Li electrode cross-section after plating (8 mAh cm⁻²) in LNBG electrolyte.


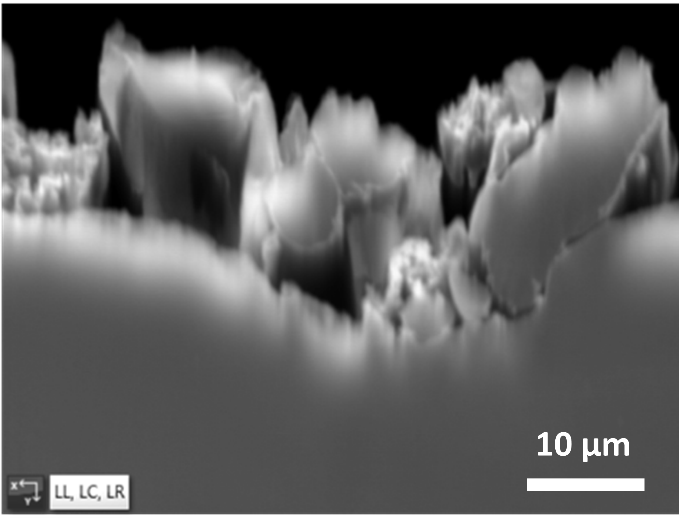


**Figure S22.** FSD image of Li electrode cross-section after plating (12 mAh cm⁻²) in LNBG electrolyte.


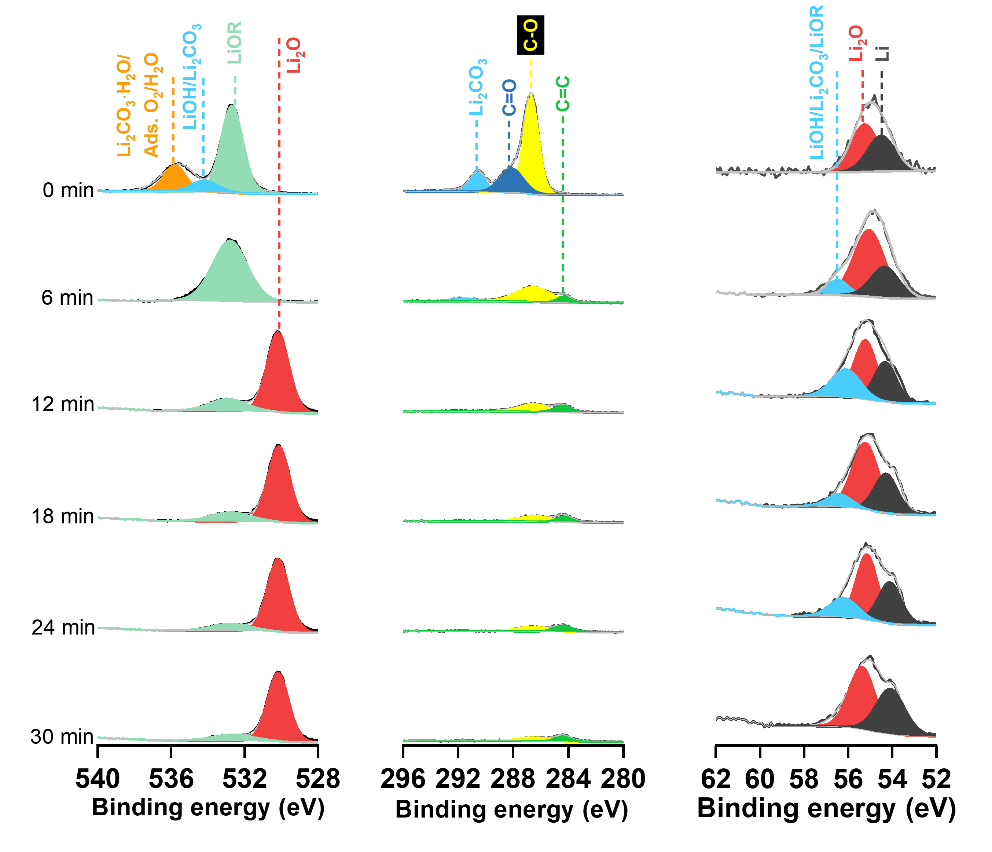


**Figure S23.** XPS of Li electrode after stripping (12 mAh cm⁻²) in LNBG electrolyte.


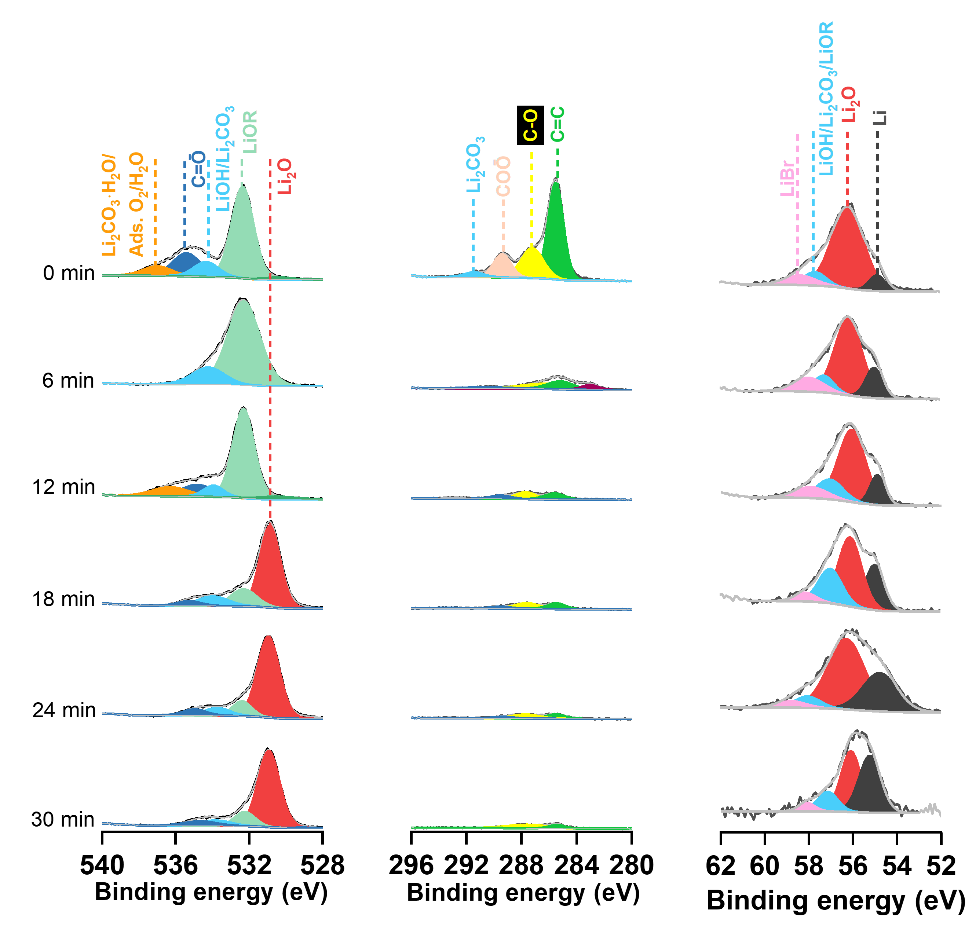


**Figure S24.** XPS of Li electrode after plating (4 mAh cm⁻²) in LNBG electrolyte.


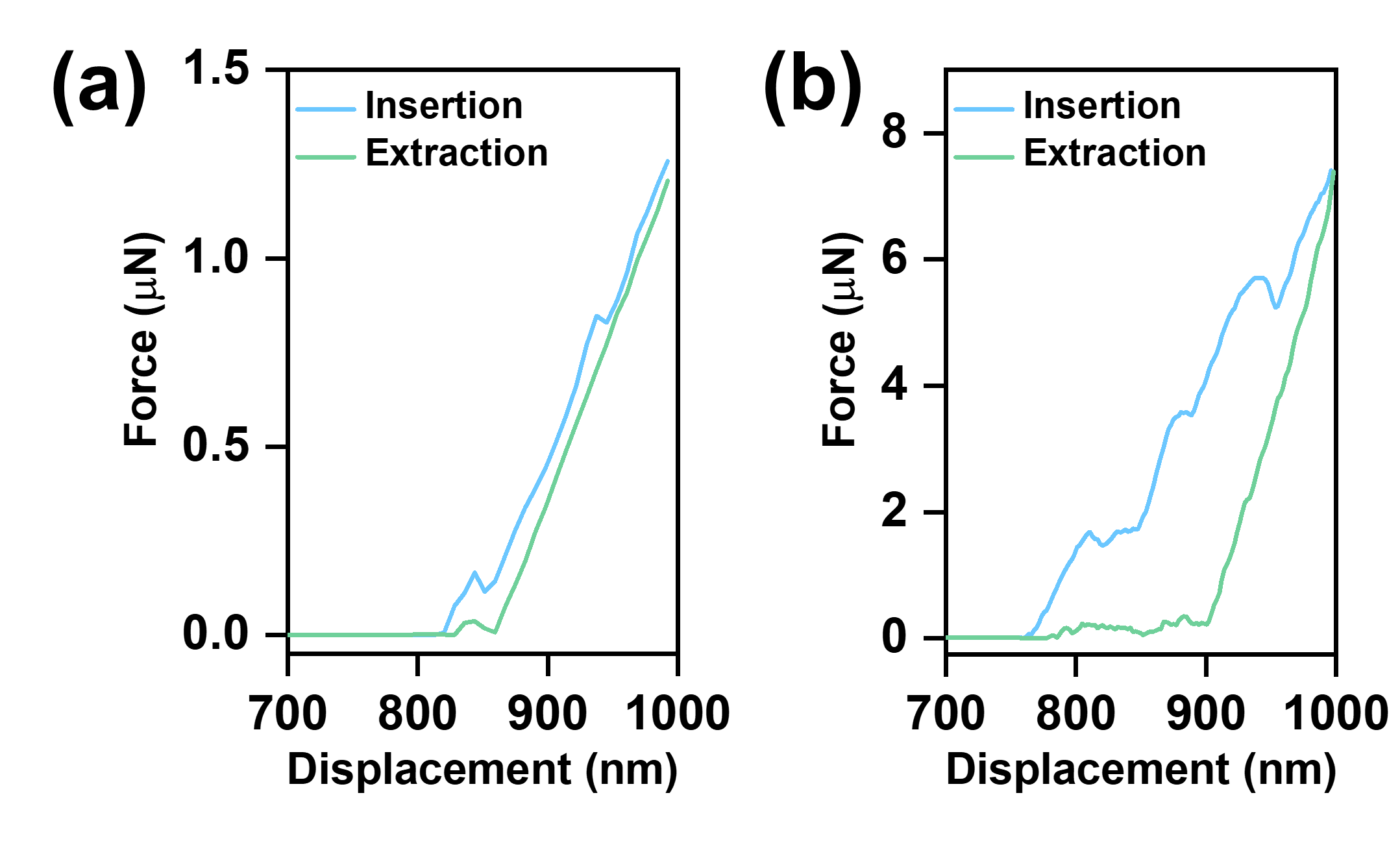


**Figure S25.** Force-displacement curves of the Li electrodes after (a) 12 mAh cm⁻² stripping and (b) 4 mAh cm⁻² plating following the stripping.


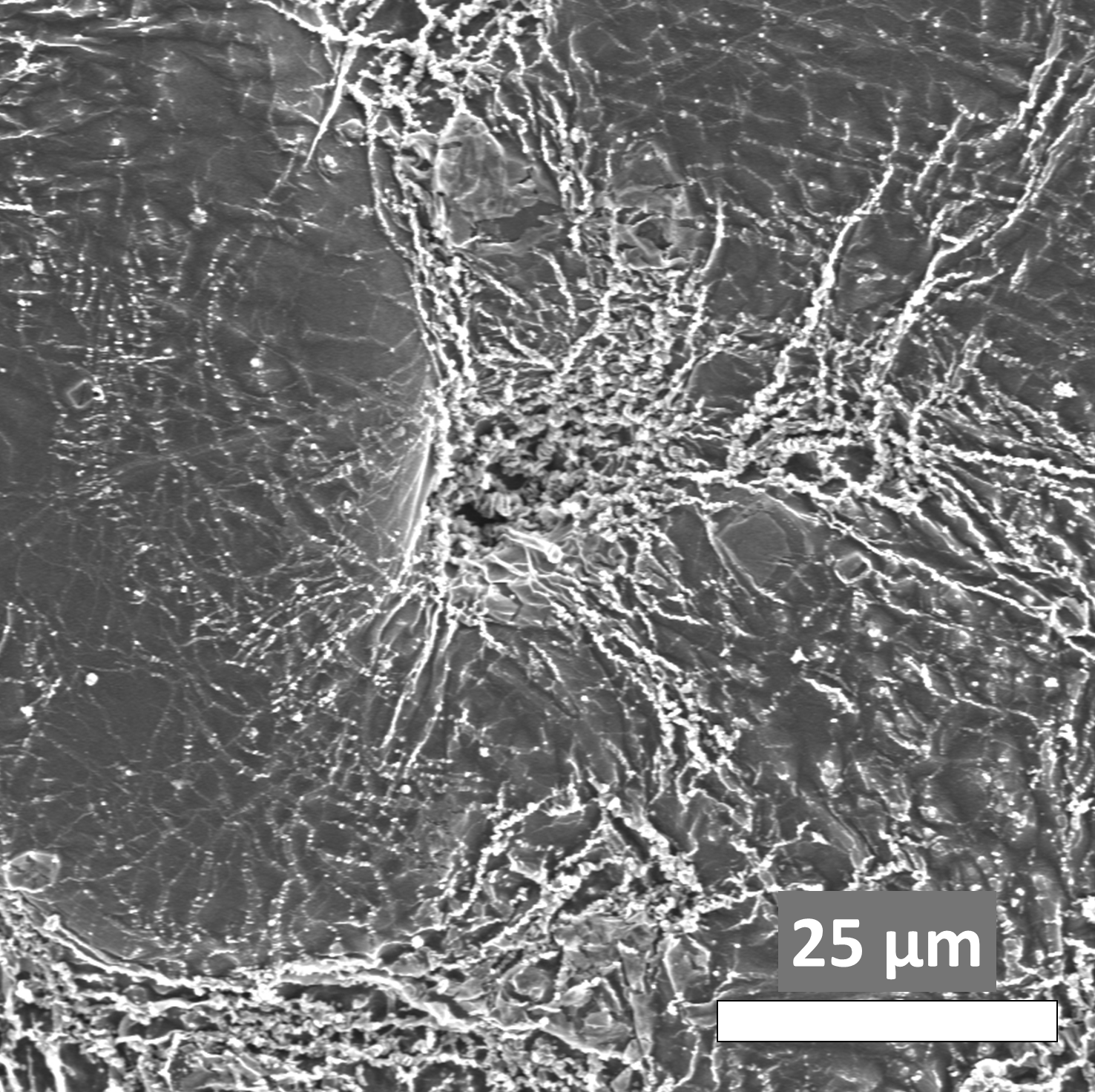


**Figure S26.** SEM of Li electrode after 3 stripping cycles (4 mAh cm⁻²) in LNBG electrolyte.


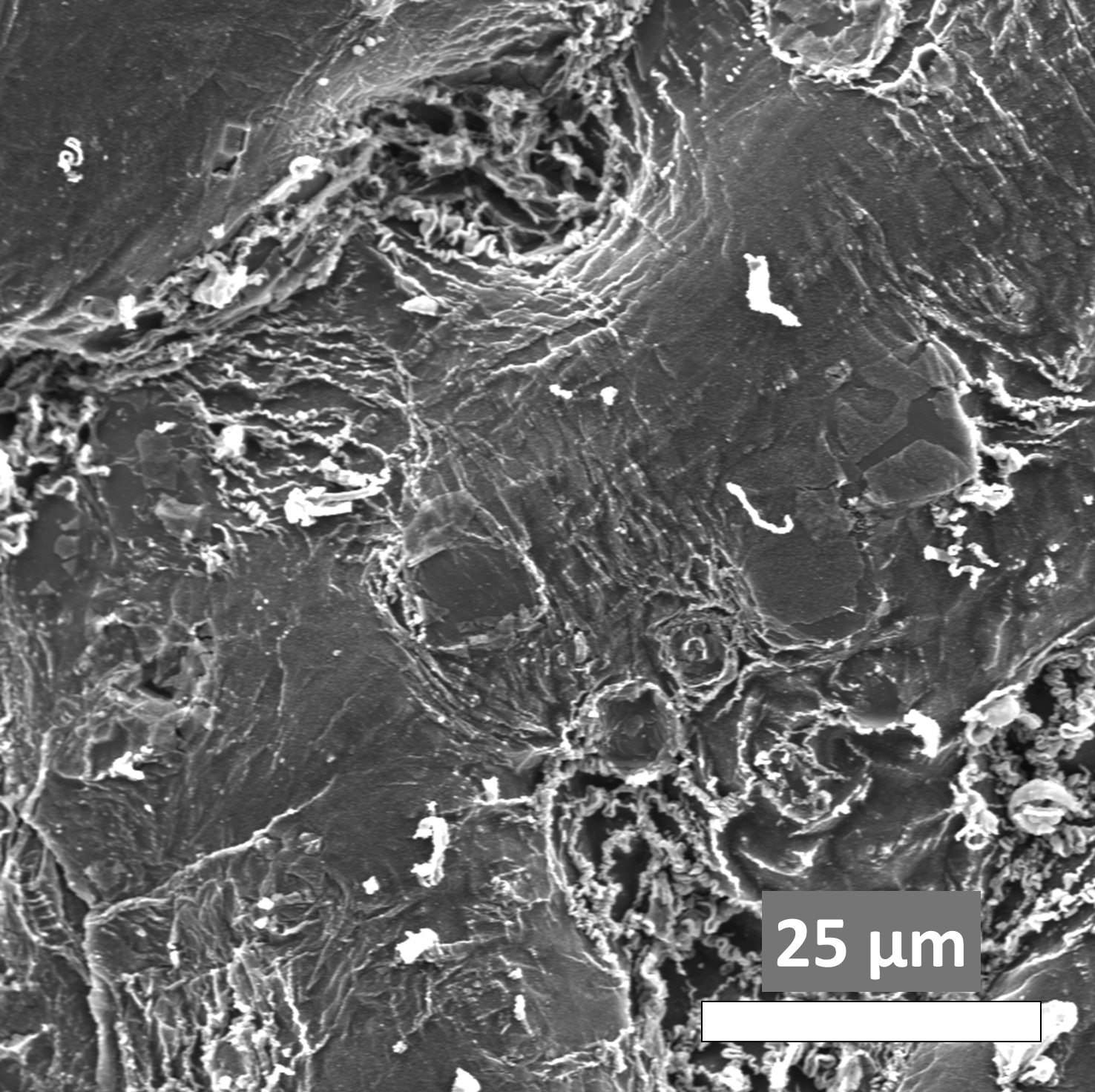


**Figure S27.** SEM of Li electrode after 6 stripping cycles (4 mAh cm⁻²) in LNBG electrolyte.


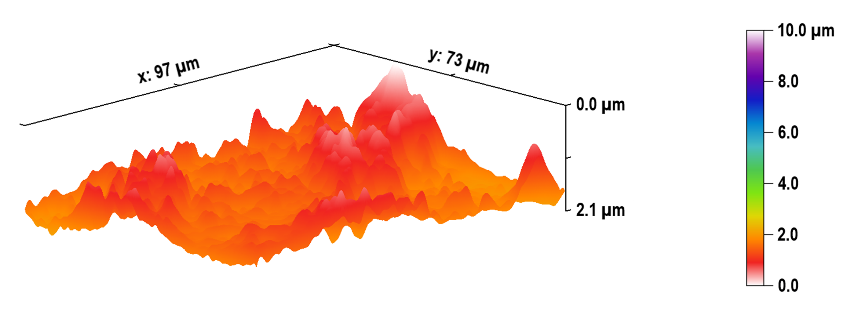


**Figure S28.** Topographic image of Li electrode after 3 stripping cycles (4 mAh cm⁻²) in LNBG electrolyte.


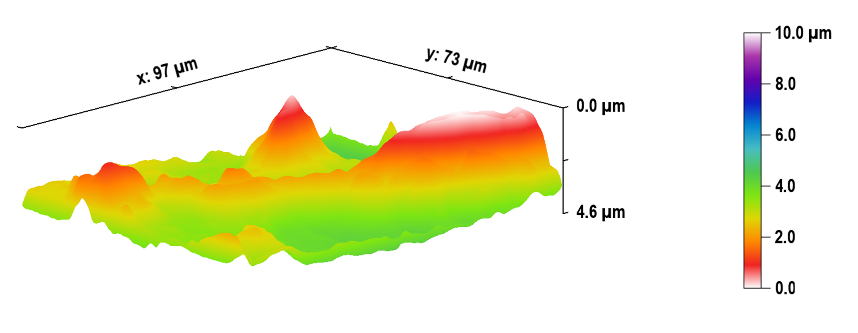


**Figure S29.** Topographic image of Li electrode after 6 stripping cycles (4 mAh cm⁻²) in LNBG electrolyte.


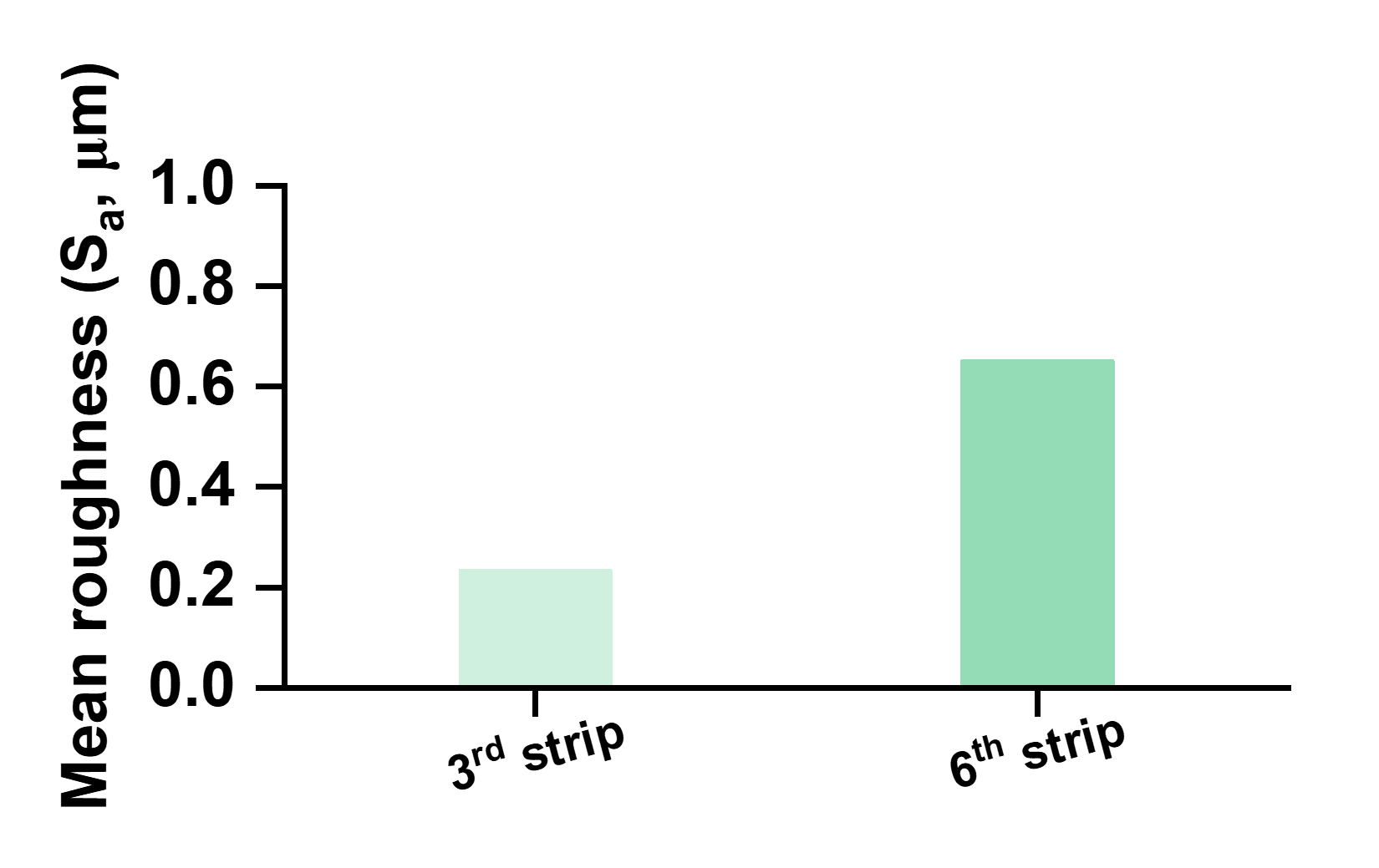


**Figure S30.** Mean roughness (S_a_) values of the Li electrodes after 3 and 6 stripping cycles (4 mAh cm⁻²) in LNBG electrolyte.


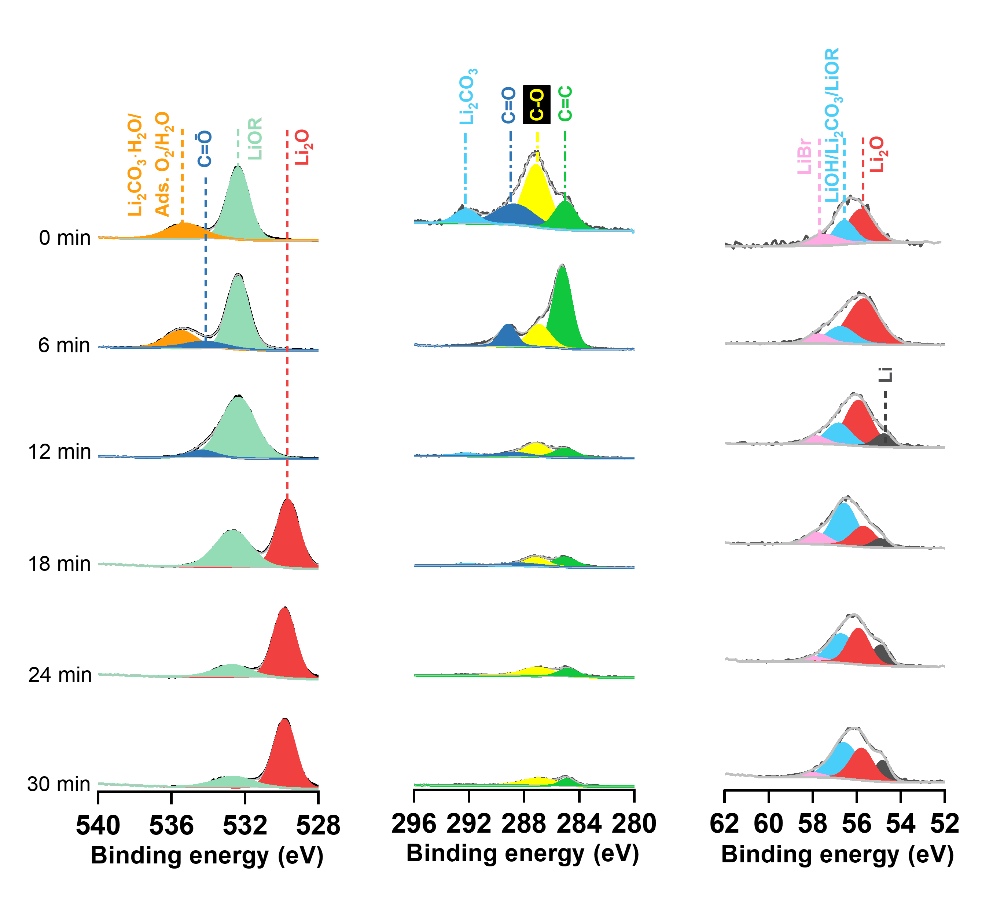


**Figure S31.** XPS of Li electrode after 3 stripping cycles (4 mAh cm⁻²) in LNBG electrolyte.


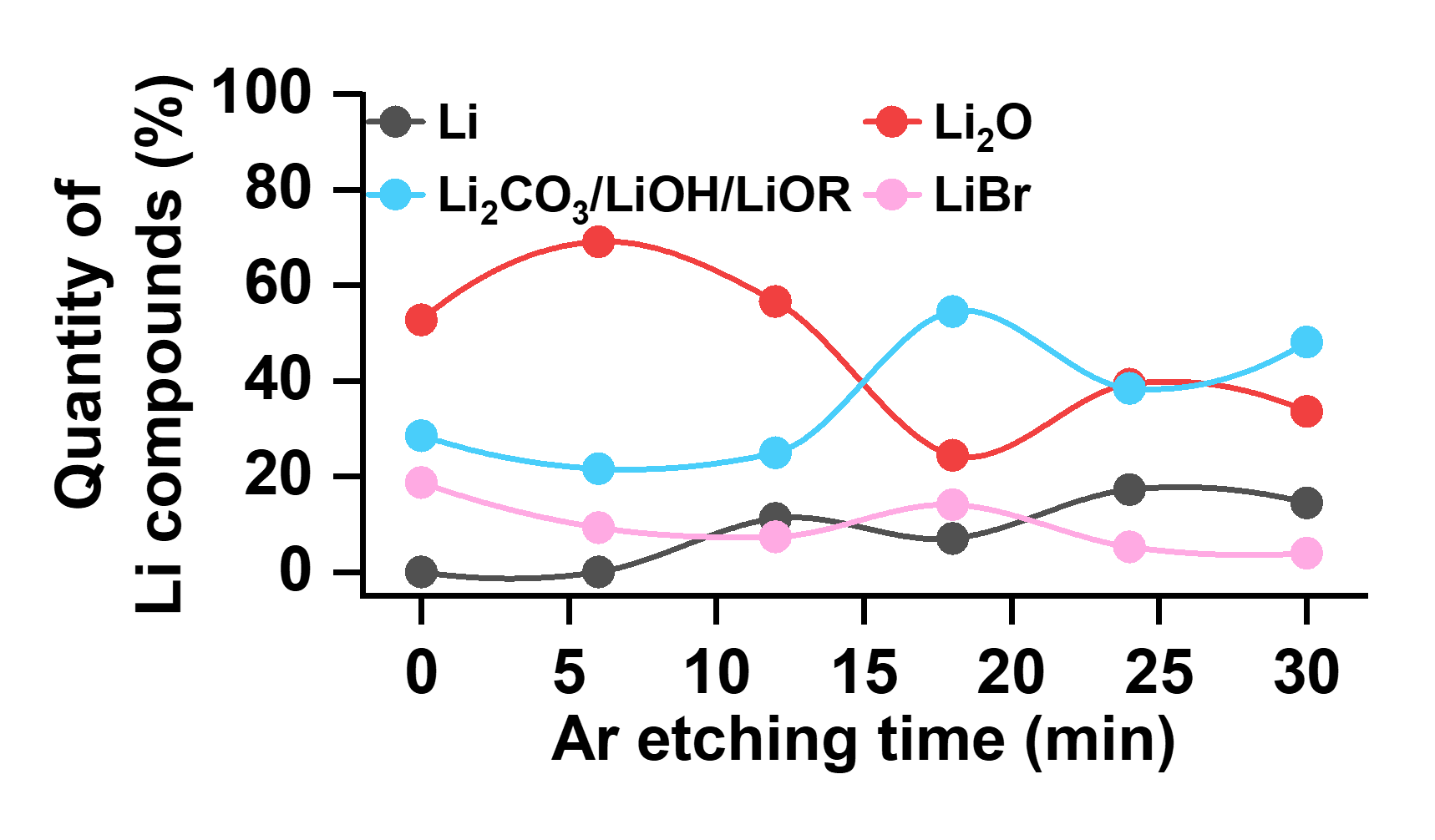


**Figure S32.** Quantification of Li compounds from the XPS of Li electrode after 3 stripping cycles (4 mAh cm⁻²) in LNBG electrolyte.
